# *Boswellia* Essential Oil: Natural Antioxidant as an Effective Antimicrobial and Anti-Inflammatory Agent

**DOI:** 10.3390/antiox12101807

**Published:** 2023-09-27

**Authors:** Diana Obiștioiu, Anca Hulea, Ileana Cocan, Ersilia Alexa, Monica Negrea, Iuliana Popescu, Viorel Herman, Ilinca Merima Imbrea, Gabriel Heghedus-Mindru, Mukhtar Adeiza Suleiman, Isidora Radulov, Florin Imbrea

**Affiliations:** 1 Faculty of Agriculture, University of Life Sciences “King Michael I” from Timisoara, Calea Aradului 119, 300645 Timisoara, Romania; dianaobistioiu@usvt.ro (D.O.); iuliana_popescu@usvt.ro (I.P.); isidora_radulov@usvt.ro (I.R.); florin_imbrea@usvt.ro (F.I.); 2 Faculty of Veterinary Medicine, University of Life Sciences “King Michael I” from Timisoara, Calea Aradului 119, 300645 Timisoara, Romania; viorelherman@usvt.ro; 3 Faculty of Food Engineering, University of Life Sciences “King Michael I” from Timisoara, Calea Aradului 119, 300645 Timisoara, Romania; ersiliaalexa@usvt.ro (E.A.); monicanegrea@usvt.ro (M.N.); gabrielheghedus@usvt.ro (G.H.-M.); 4 Faculty of Engineering and Applied Technologies, University of Life Sciences “King Michael I” from Timisoara, Calea Aradului 119, 300645 Timisoara, Romania; ilinca_imbrea@usvt.ro; 5 Faculty of Life Science, Department of Biochemistry, Ahmadu Bello University, Zaria 810107, Kaduna State, Nigeria; masuleiman@abu.edu.ng

**Keywords:** *Boswellia carteri*, *Boswellia sacra*, *Boswellia papryfera*, *Boswellia frereana*, *Boswellia* essential oil, chemical composition, antioxidant, anti-inflammatory, antimicrobial activity, molecular docking

## Abstract

The research aimed to determine the chemical composition, the antioxidant and anti-inflammatory activity as well as the antimicrobial activity against Gram-positive, Gram-negative and two fungal *Candida* ATCC strains of a commercial *Boswellia* essential oil (BEO) containing *Boswellia carteri*, *Boswellia sacra*, *Boswellia papryfera*, and *Boswellia frereana*. Additionally, molecular docking was carried out to show the molecular dynamics of the compounds identified from the essential oil against three bacterial protein targets and one fungal protein target. The major components identified by GC-MS (Gas Chromatography-Mass Spectrometry) were represented by α-pinene, followed by limonene. Evaluation of antioxidant activity using the DPPH (2,2-Diphenyl-1-Picrylhydrazyl) method showed high inhibition comparable to the synthetic antioxidant used as a control. Oxidative stability evaluation showed that BEO has the potential to inhibit primary and secondary oxidation products with almost the same efficacy as butylated hydroxyanisole (BHA). The use of BEO at a concentration of 500 ppm provided the best protection against secondary oxidation during 30 days of storage at room temperature, which was also evident in the peroxide value. Regarding the in vitro anti-inflammatory activity, the membrane lysis assay and the protein denaturation test revealed that even if the value of protection was lower than the value registered in the case of dexamethasone, the recommendation of using BEO as a protective agent stands, considering the lower side effects. Gram-positive bacteria proved more sensitive, while *Pseudomonas aeruginosa* presented different sensitivity, with higher MICs (minimal inhibitory concentration). *Haemophilus influenzae* demonstrated a MIC at 2% but with consecutive inhibitory values in a negative correlation with the increase in concentration, in contrast to *E. coli*, which demonstrated low inhibitory rates at high concentrations of BEO. The computational tools employed revealed interesting binding energies with compounds having low abundance. The interaction of these compounds and the proteins (tyrosyl-tRNA synthetase, DNA gyrase, peptide deformylase, 1,3-β-glucan synthase) predicts hydrogen bonds with amino acid residues, which are reported in the active sites of the proteins. Even so, compounds with low abundance in BEO could render the desired bioactive properties to the overall function of the oil sustained by physical factors such as storage and temperature. Interestingly, the findings from this study demonstrated the antioxidant and antimicrobial potential of *Boswellia* essential oil against food-related pathogens, thus making the oil a good candidate for usage in food, feed or food-safety-related products.

## 1. Introduction

The control and treatment of bacterial maladies, primarily caused by bacterial mutants resistant to most available antibiotics, is a real and pressing concern. Numerous studies concentrate on alternative or complementary antimicrobial strategies due to these facts. Antimicrobial compounds derived from natural resources, such as plant extracts, essential oils, and peptides, are garnering increasing interest for their activity against various microorganisms in the hope that, unlike antibiotics, they will be effective without causing resistance.

*Boswellia* is a genus of trees in the order Sapindales, which contains approximately twenty species of aromatic resin-producing plants. The biblical frankincense is an extract of the substance of *Boswellia sacra* and *Boswellia frereana*, which has found wide application in medicine. Extracts of *B. sacra* have been utilised due to their analgesic, antioxidant, cardioprotective, and anti-inflammatory properties [1,2]. In addition, the same oil can be recommended to prevent and treat certain autoimmune inflammatory diseases [3]. Another medical applicability is controlling metabolic syndrome and its associated disorders, including hyperglycemia, dyslipidemia, hypertension, obesity, and diabetes [4].

The antimicrobial activity of diverse *Boswellia* oils depends on the tested bacterial strains and their chemical composition. The climate influences the chemical compounds of *Boswellia* species, the geographical location of the plant source, the age of the tree, and the harvest season or conditions. Di Stefano et al. [5] demonstrate the various biochemical contents of *Boswellia,* three different varieties of essential oil from the Dhofar region (South Oman), grown under various agroclimatic conditions (Houjri, Najdi, and Sahli) on the eastern border of Yemen. The study revealed that Houjri essential oil, the first hydro-distillate obtained from *B. sacra*, contained the maximum concentration of volatile components (37 compounds identified) compared to Sahli (29 compounds identified) and Najdi (23 compounds identified) oil [5].

Biochemical elucidation of the identified compounds from the hydrodistillation technique can be achieved using molecular docking analysis [6]. Interestingly, this would help understand the repertoire display between the compounds and specific protein targets of the bacteria and fungi. The presence of aminoacyl-tRNA synthetases (aaRS) in living organisms applauds their involvement in the translation of genetic code [6], thus making them an important antibacterial target. Inhibiting this enzyme prevents protein synthesis and discontinuity of bacterial growth [7]. Importantly, tyrosyl-tRNA synthetase (TyrRS) is a family of aaRS that are highly conserved in prokaryotes and have been reported as a possible target in therapy that uses antibiotics [8]. The crystal structure of TyrRS protein has been resolved to 420 amino acids (PDB: IJIJ). Recent research by Alminderej et al. [9] demonstrated that certain monoterpenes and sesquiterpenes constituents of essential oils inhibit cell membrane permeability along with the potential binding modes against tyrosyl-tRNA synthetase (TyrRS) enzyme of bacteria [9]. In addition, DNA gyrase (PDB ID: 1AB4) is an interesting antibacterial target commonly involved in testing first-line drugs for bacterial infections. The enzyme belongs to the type II topoisomerase and is needed during bacterial cell division for supercoiling chromosomal DNA [10]. Moreover, drugs and antibiotics attempt to inhibit this enzyme’s catalytic function, thereby causing fragmentation of the genomic material [11]. The involvement of peptide deformylase (PDF) in the maturation of emerging polypeptides during protein biosynthesis has made this enzyme an important target for antibacterial agents [12]. The formylation and deformylation cycle are conditional for successful bacterial growth [13]. Thus, inhibitors of PDF are reported to show promising progress in both in vitro and in vivo assessment, leading to impairment of the deformylation of multiple bacterial proteins [14]. Peptide deformylase protein ID: 1IX1 has 171 amino acid residues. The protein target, 1,3-β-glucan synthase, is a glucosyltransferase enzyme involved in the biosynthesis of fungal cell wall structure 1,3-β-glucan [15]. Furthermore, this protein is important for growth and morphogenesis in fungi as well as a potential *Candida* species inhibition target by most antifungal agents [16]. The protein (PDB ID: 7XE4) is 1876 amino acids sequence in length.

*Boswellia* oils distributed by various corporations on the global market come from various sources. The chemical differences between products on the market influence their antimicrobial activity, causing some to be more effective than others [5,17,18]. The objectives of this research were to determine the chemical composition of GC-MS (Gas Chromatography-Mass Spectrometry), antioxidant potential by DPPH (2,2-Diphenyl-1-Picrylhydrazyl), PV (Peroxide Index), p-AV (p-Anisidine Value), TOTOX (Total Oxidation Value) and TBA (Thiobarbituric acid), anti-inflammatory potential, effect on protein denaturation, antimicrobial activity against Gram-positive, Gram-negative bacteria and two *Candida* strains, evaluation complemented by molecular docking to show the molecular dynamics of the compounds identified from the essential oil against the TyrRS, DNA gyrase, peptide deformylase proteins of the tested bacterial strains and the 1,3-β-glucan synthase of the fungi strains for supporting the promising inhibitory potential of compounds as predicted by the binding energies.

## 2. Materials and Methods

### 2.1. Chemicals

Ethanol (Sigma–Aldrich; Merck KGaA, Darmstadt, Germany), 1,1-diphenyl-2-picrylhydryl (DPPH, Sigma–Aldrich, Taufkirchen, Germany), isooctane (Sigma–Aldrich Chemie GmbH, Munich, Germany), glacial acetic acid (Sigma–Aldrich Chemie GmbH, Munich, Germany), benzene (Sigma–Aldrich Chemie GmbH, Munich, Germany), thiobarbituric acid (Sigma–Aldrich Chemie GmbH, Munich, Germany), PBS isotonic solution (RemedLab, Bucharest, Romania) and egg albumin (Oxford Lab Fine Chem, Maharashtra, India).

All reagents used for chemical analysis were purchased from Sigma–Aldrich Chemie GmbH (München, Germany) and Geyer GmbH (Renningen, Germany) and were of analytical quality.

#### 2.1.1. Oil Samples

The essential oil used in the present study (BEO) is a commercial oil (Lot 2032310/2020, doTERRA, Pleasant Grove, UT, USA), a mixed product from *B. carteri*, *B. sacra*, *B. papryfera* and *B. frereana* obtained through steam distillation. The essential oil was stored per the producer’s recommendations in room-temperature conditions, avoiding direct sunlight or prolonged exposure to oxygen using tightly secured caps for the duration of one year.

Sunflower oil (SFO) was purchased from Solaris (Bucharest, Romania), which produces cold-pressed vegetal oils, natural, 100%, without adding any additives.

#### 2.1.2. Gas Chromatography-Mass Spectrometry (GC/MS)

GC-MS analysed BEO using Shimadzu QP 2010 Plus apparatus (Columbia, SC, USA) equipped with an AT WAX 30 m 0.32 mm 1 µm capillary column. The discharge rate of the carrier gas, helium, was 1 mL/min, and the temperatures of the injector and ion source were 250 °C and 220 °C, respectively. For compound separation, a temperature gradient was utilised with an initial oven temperature of 40 °C maintained for 1 min, followed by an increase to 210 °C at a rate of 5 °C/min and a subsequent 5-min hold at this temperature. The sample injection volume was 1 μL of a 2% BEO hexane solution, and a split ratio of 1:50 was utilised. The GC-MS analysis was executed in triplicate.

The volatile components of the essential oil evaluated were identified using the NIST 5 Wiley 275 library database. The match of detected compounds to the database was a minimum of 90%. The results were presented as percentages from total compounds. LRI (Linear Retention Index) was calculated using Normal alkane RI for the same polar column [19]. The values obtained refer to the percentage area of the chromatographic bands (peaks) on the chromatogram corresponding to the compounds identified.

### 2.2. Antioxidant Capacity by 1,1-Diphenyl-2-Picrylhydrazyl (DPPH) Assay

The determination of the antioxidant capacity using the DPPH method was carried out according to the method described by Ciulca et al. with minor modifications [20]. To characterize the antioxidant characteristics of BEO, an alcoholic extract was prepared. For this purpose, 1 mL of BEO was dissolved in 10 mL of methanol (Sigma–Aldrich; Merck KGaA, Darmstadt, Germany) using an ultrasonic water bath (FALC Instruments, Treviglio, Italy) for 30 min at room temperature. Butylated hydroxytoluene (BHT) (200 ppm concentration) was used as a control sample and methanol was used as a negative control. After 30 min of stirring, the extracts were filtered through Whatman filters fitted with a 0.45 µm nylon membrane of 30 mm diameter (Sigma–Aldrich; Merck KGaA, Darmstadt, Germany). The filtered extracts were stored at 2–4 °C until analysis.

Dilutions of 5 different concentrations (50; 60; 70; 80 and 100 ug/mL) were made from the base extract. 3 mL of each dilution was taken, and 1 mL of 0.1 mM DPPH was added to each dilution. The samples thus prepared were left to stand in the dark for 30 min. The absorbance of the samples was read at a wavelength of 518 nm using a UV-VIS spectrophotometer (Specord 205; Analytik Jena AG, Jena, Germany). Three determinations were made for each extract, and the result was reported as the mean value.

A control sample was performed in parallel, in which the extract was replaced by a mixture of distilled water and DPPH solution in the same volume and concentration.

Antioxidant activity was calculated as a percentage of Radical Scavenging Activity (RSA) according to Formula (1):

(1) $$\mathrm{RSA}\;(\%)=\frac{A_{control}\;-\;A_{samples}}{A_{control}}\cdot 100$$

where

*A_control_*—the absorbance value of the control sample
*A_sample_*—the absorbance values of the extracted sample.

The antioxidant capacity of the extracts was expressed as IC50 value and compared with that of ascorbic acid.

### 2.3. Oxidative Stability Determination

#### 2.3.1. Application of BEO to Sunflower Oil

6 samples of 50 mL each of SFO without synthetic antioxidants were prepared in vials with caps. In the first, 200 ppm (*w*/*v*) butylated hydroxytoluene (BHT) (the maximum legal permitted dose) was added; in four of the six SFO containers, different proportions of pure BEO were added, i.e., 100 ppm, 200 ppm, 300 ppm and 500 ppm (*v*/*v*), and the sixth sample containing only SFO was used as a control sample. The sample containers were shaken for 30 min at room temperature using a mechanical shaker (Heidolph, Wood Dale, IL, USA) for complete homogenisation. The oil sample containers were stored for 30 days in the dark at room temperature (25 °C). Each oil sample was prepared in triplicate, and then at 5-day intervals, samples were taken to assess oxidative stability by determining peroxide value (PV), p-anisidine (p-AV), TOTOX value and thiobarbituric acid (TBA) value.

#### 2.3.2. Determination of Peroxide Value

According to the accepted oil analysis procedure, the peroxide index (PV) was determined using the iodometric method, and the results were expressed in meq O_2_/kg oil.

The samples were prepared by homogenising them with a chopstick, preventing the oil from aerating. From the prepared sample, 1 g of oil was weighed to the nearest 0.001 g into a bottle with a ground glass stopper. Chloroform was added over 10 mL and shaken until dissolved. Then, 15 mL of glacial acetic acid and 1 mL of saturated potassium iodide solution were added. The flask was immediately closed, shaken for one minute and allowed to stand for 5 min in the dark. After the 5 min had elapsed, 75 mL of distilled water was added. The released iodine was titrated in the presence of 1% starch solution as an indicator by vigorous shaking with 0.01 n sodium thiosulphate solution.

A control sample (without the product to be analysed) was carried out in parallel.

The peroxide value, expressed in milli-equivalents of peroxide per 1 kg of product, was calculated using the formula:

(2) $$Peroxide\;value=\frac{\left(V_{1}-V_{2}\right)\cdot n}{m}\cdot 1000\;(meq/kg)$$

where:

*V*_1_—volume of sodium thiosulphate solution in the titration of the test sample (mL);
*V*_2_—volume of sodium thiosulphate solution in the titration at the blank determination, (mL);
*m*—mass of analyte sampled in the blank determination, (g);
*n*—normality of the sodium thiosulphate solution used in the titration (0.01 respectively) [21].

#### 2.3.3. p-Anisidine Value (p-AV)

The p-AV was determined according to the official spectrophotometric method (AOCS Official Method Cd 18-90) [22]. From each oil sample prepared as described in Section 2.3.1, 2 g was taken. 25 mL of isooctane was added and shaken manually for a few seconds to homogenise. Immediately, the absorbance was read at 350 nm against an isooctane sample using a UV-VIS double-beam spectrophotometer (Specord 205; Analytik Jena AG, Jena, Germany). From the previously prepared solutions, 5 mL were taken and placed in separate containers with 1 mL of 0.25% *w/v* p-anisidine/glacial acetic acid solution. After 10 min, the absorbance was read again at 350 nm using a UV-Vis spectrophotometer (Specord 205; Analytik Jena AG, Jena, Germany). The p-AV value was calculated using Equation (3):

(3) $$\mathrm{p-AV}=25\;\times \;\frac{1.2\times A_{2}-A_{1}}{W}$$

where

*A*_1_—absorbance of oil samples dissolved in isooctane;
*A*_2_—absorbance of oil samples in isooctane and p-anisidine solution;
*W*—mass of sunflower oil samples (g).

#### 2.3.4. Total Oxidation Value (TOTOX)

The TOTOX value represents the degree of total oxidation and was calculated based on the PV and p-AV values previously determined according to Equation (4) [20]:

TOTOX value = 2·PV + p-AV(4)

#### 2.3.5. Assessing the Lipid Oxidation Degree of Oil Samples by Thiobarbituric Acid (TBA) Test

The TBA test was also carried out according to the method described by Cocan et al. [23], with slight modifications, to confirm the protective effect of BEO against SFO oxidation. Thus, 2 g were weighed from each sample, and 5 mL benzene and 4 mL thiobarbituric acid (0.67% aqueous solution) were added. The resulting mixture was shaken using a mechanical shaker (Heidolph, Illinois, IL, USA) for 30 min at room temperature, after which the samples were allowed to stand for 10 min for phase separation. The supernatant was collected from each sample in separate containers and heated in a water bath at 80 °C for 45 min. After cooling the samples, the absorbance was read at 540 nm using a spectrophotometer (Specord 205; Analytik Jena AG, Jena, Germany) against a control sample prepared according to the above procedure, but in which no oil was introduced. The calibration curve was performed using malonaldehyde (MDA) in the 0–50 g malonaldehyde (MDA)/g concentration range. The results were expressed in g (MDA)/g oil.

### 2.4. Anti-Inflammatory Activity

#### 2.4.1. Membrane Lysis Assay

##### Preparation of Red Cell Suspension

The procedure outlined by Gunathilake et al. [24] was used to prepare the erythrocyte suspension with slight modifications. Human heparinised blood was centrifuged at 3000 rpm for 10 min. After centrifugation, the supernatant was removed, and the erythrocyte mass was washed with an equal volume of isotonic sodium chloride solution (0.9%). The centrifugation and washing steps were repeated three times. Subsequently, the blood volume was measured and reconstituted as 40% suspension with isotonic PBS solution at a pH of 7.4.

#### 2.4.2. Heat-Induced Haemolysis

The heat-induced haemolysis assay was conducted in accordance with the method developed by Okoli et al. [25], with some modifications exposed by Gunathilake et al. [24]. Briefly, different concentrations of essential oil (10 µL/mL, 20 µL/mL, 40 µL/mL, 80 µL/mL, 160 µL/mL) were suspended in 5 mL of PBS isotonic solution (RemedLab, Bucharest, Romania) at pH 7.4, over which 100 µL red blood cell suspension was added. After delicate shaking, the samples were incubated in a water bath at 54 °C for 20 min. The samples were centrifuged at 2500 rpm for 3 min at the end of the incubation period, and the absorbance of the supernatant was measured at 540 nm using a UV-VIS spectrophotometer (Specord 205; Analytik Jena AG, Jena, Germany). The negative control sample consisted of PBS and 100 µL erythrocyte suspension. The positive control sample consisted of 0.1 mg/mL of dexamethasone diluted in 5 mL of PBS and 100 µL erythrocyte suspension.

Formula (5) was used to determine the percentage of haemolysis inhibition:

(5) $$\%\;inhibition\;of\;haemolysis=100-\frac{A_{1}}{A_{2}}*100$$

where:

*A*_1_ represents the absorbance of the tested sample
*A*_2_ represents the absorbance of the negative control.

#### 2.4.3. The Effect on Protein Denaturation

The protein denaturation assay was executed according to the method described by Gunathilake et al. [17], with slight modifications. Different concentrations of tested essential oil (10 µL/mL, 20 µL/mL, 40 µL/mL, 80 µL/mL, 160 µL/mL) were each added to 1 mL of 1% egg albumin (Oxford Lab Fine Chem, Maharashtra, India) and 4 mL of PBS with an acid pH (pH 6.4) (RemedLab, Bucharest, Romania). The solution was incubated for 15 min at 37 °C and then heated at 70 °C for 5 min in a water bath (D-91126, Memmert GmbH & Co. KG, Schwabach, Germany). After cooling, the absorbance was read at 660 nm using a UV-VIS spectrophotometer (Specord 205; Analytik Jena AG, Jena, Germany).

The control solution was a mixture of albumin and PBS without essential oil.

The percentage inhibition of protein denaturation was calculated using the Formula (6):

(6) $$\%\;inhibition=100-\frac{A_{1}}{A_{2}}*100$$

where:

*A*_1_ represents the absorbance of the tested sample
*A*_2_ represents the absorbance of the control.

### 2.5. Antimicrobial Activity

The antimicrobial activity of BEO was determined by broth microdilution against Gram-positive and Gram-negative bacteria and two *Candida* fungal ATCC strains.

The ATCC strains utilised in this study were obtained from the University of Life Sciences “King Michael I of Romania” Timişoara’s Laboratory of Microbiology culture collection, part of the Interdisciplinary Research Platform. The tested strains were: *S. pyogenes* (ATCC 19615), *S. aureus* (ATCC 25923), *L. monocytogenes* (ATCC 19114), *Cl. perfringens* (ATCC 13124), *B. cereus* (ATCC 10876), *S. flexneri* (ATCC 12022), *P. aeruginosa* (ATCC 27853), *E. coli* (ATCC 25922), *S. typhimurium* (ATCC 14028), *H. influenzae* type B (ATCC 10211), *C. albicans* (ATCC 10231), and *C. parapsilopsis* (ATCC 22019).

#### 2.5.1. Bacterial Culture

Our previous study describes the methods [26,27]. The BEO was used directly by adding 2, 4, 8, 16, or 32 µL over the bacterial suspension equivalent of 20, 40, 80, 160, or 320 mg/mL. Pure uninhibited strain in Brain Heart Infusion Broth (BHI) (Oxoid, CM1135) was used as a negative control, and the value was subsequently used to calculate the bacterial growth and inhibition rates.

The MIC was determined by measurement of OD using the spectrophotometric method, according to ISO 20776-1:2019. The MIC is the lowest sample concentration tested at which there is no discernible growth of microorganisms. BGR (bacterial growth rate) and BIR (bacterial inhibition rate) were calculated as indicators for interpreting the results using the following Formulas (7) and (8):

(7) $$\mathrm{BGR}=\frac{OD_{sample}}{OD_{negative\;control}}\times 100\;(\%)$$

BIR = 100 − BGR (%)(8)

where:

OD_sample_—optical density at 540 nm as a mean value of triplicate readings for essential oil in the presence of the selected bacteria;
OD_negative control_—optical density at 540 nm as a mean value of triplicate readings for the selected bacteria in BHI.

The IC50 value calculated using the mean average OD values obtained for each BEO concentration and strain tested denotes the concentration at which BEO demonstrates 50% of its maximum inhibitory effect.

#### 2.5.2. Fungal Culture

The analysis of *C. parapsilopsis* and *C. albicans* was executed according to our previous research [28], with small modifications regarding the oil quantity tested. BEO was placed directly at 2 µL, 4 µL, 8 µL, 16 µL and 32 µL into a 96 microdilution well plate, each well achieving a concentration of 2%, 4%, 8%, 16% and 32% or an equivalent of 20, 40, 80, 160 or 320 mg/mL. The plates were incubated at 37 °C for 48 h. After incubation, the OD was measured at 540 nm. All samples were read in triplicate.

The following formulas were used to calculate MGR (mycelial growth rate) (9) and MIR (mycelial inhibition rate) (10):

(9) $$\mathrm{MGR}=\frac{OD_{sample}}{OD_{negative\;control}}\times 100\;(\%)$$

MIR = 100 − MGR (%) (10)

where: OD_sample_—a mean value of triplicate readings for essential oil in the presence of the selected fungi at 540 nm; OD_negative control_—a mean value of triplicate readings for the selected fungi in BHI.

### 2.6. Molecular Docking Study

The crystal structure of bacterial tyrosyl-tRNA synthetase (PDB ID: 1JIJ) DNA gyrase (PDB ID: 1AB4), peptide deformylase (PDB ID: 1IX1) and fungal 1,3-β-glucan synthase (PDB ID: 7XE4) were retrieved from RCSB Protein Data [29]. At the same time, the 3D SDF file of the thirty-six (36) compounds was downloaded from the PubChem database [30]. Chimera docking software version 1.15 was used to prepare the protein for docking [31]. During the docking preparation, the co-crystallised ligand was removed, leaving the free protein and dock prep was performed by deleting water molecules, adding polar hydrogens and Gasteiger charges were assigned. After that, a PDB format was saved for the prepared protein. The docking procedure for the bacterial protein was initiated following command to the Autodock Vina wizard to commence the docking, and the grid boxes centre (x, y, z coordinates −12.765, 17.408, 82.113) and size (x, y, z coordinates 25.454, 27.326, 54.111) for 1JIJ, (x, y, z coordinates 67.690, 84.102, 49.786) and size (x, y, z coordinates 41.322, 46.896, 84.272) for 1AB4, (x, y, z coordinates 56.273, 82.802, 2.963) and size (x, y, z coordinates 39.625, 42.644, 34.137) for 1IX1, while for the fungi, the grid boxes centre (x, y, z coordinates 100.714, 85.871, 122.709) and size (x, y, z coordinates 92.145, 66.057 86.716) to cover the binding site of the active pocket of the protein. Upon the completion of the docking, files from PDB to PDBQT were converted, and the lowest binding energy of the compounds and the protein was recorded.

Receptor-ligand interaction was finally visualised in Discovery Studio Visualizer [32]. This was required to ascertain the potential bonding connections between the amino acid residues of the tyrosyl-tRNA synthetase, DNA gyrase, peptide deformylase proteins and the compounds, as well as between the amino acid residues of 1,3-β-glucan synthase and the compounds of *Boswellia* oil respectively.

### 2.7. Statistical Analysis

All measurements were taken in triplicate, and the findings are given as mean values with standard deviation (SD). Statistical processing data, including IC50, was performed using Microsoft Excel 365. Data were analysed by one-way analysis of variance (ANOVA) to assess if the addition of BHT and BEO represents a source of variance related to measured parameters.

## 3. Results

### 3.1. Gas Chromatography-Mass Spectrometry (GC/MS)

The GC-MS analysis revealed 36 compounds, chemical constituents above 0.5% present in the composition of BEO in Table 1. The values obtained refer to the percentage area of the chromatographic bands (peaks) on the chromatogram corresponding to the compounds identified. The full table of constituents is presented as a Supplementary File S1.

Monoterpene hidrocarbonates (MH) represented the majority chemotypes (71.49%), followed by Monoterpene oxygenate (MO) (12.83%) and sesquiterpene hidrocarbonates (SH) (3.02%). Sesquiterpene oxygenate (SO) was not identified in the chromatographic profile of the analysed essential oil.

The major component of the studied essential oil is represented by α-pinene, at a concentration of 39.34%, followed by limonene (13.79%). Except for α phellandrene (5.48%), the other compounds were found at a concentration under 5%. The values of the rest of the chemical compounds were under 1%.

The producer has available the quality control analysis by GC/MS using a Shimadzu GCMS-QP2010 Ultra on the website [34], the results obtained being similar to the present research.

### 3.2. Antioxidant Profile

The antioxidant activity (DPPH) of BEO is presented in Table 2. A synthetic antioxidant sample (BHT) was also analysed to highlight the antioxidant effect of BEO.

The radical scavenging activity by the DPPH method of BEO was determined for 5 concentrations (50 µg/mL, 60 µg/mL, 70 µg/mL, 80 µg/mL and 100 µg/mL) (Table 3). In parallel, the antioxidant activity of 5 solutions of ascorbic acid in different concentrations (0.06–0.16 µg/mL) was evaluated as a positive control, resulting in an inhibition of 94.54% for the highest concentration tested (0.16 mg/mL). The IC50 (concentration of each dilution resulting in 50% DPPH inhibition) was subsequently calculated and expressed in µg/mL (Table 4).

Table 4 shows the values obtained for IC50 compared to the value obtained for the control sample, ascorbic acid.

### 3.3. Oxidative Stability

#### 3.3.1. Peroxide Value

**Figure 1:**
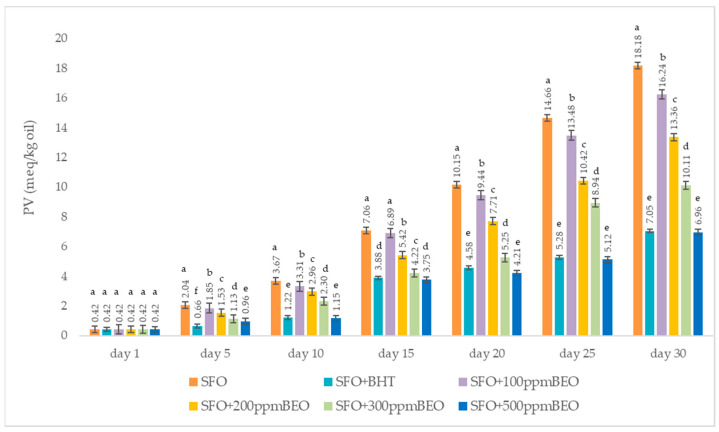
Peroxide value of oil samples The values are given as an average ± standard deviations of three separate determinations. a–f ANOVA The test was used to examine the average differences across samples taken on the same day; mean values marked in different lowercase letters in ascending order show significant differences between the samples on the same day (*p* < 0.05); average values marked in identical lowercase letters show that there are no significant differences between samples on the same day (*p* < 0.05).

Figure 1 expresses the changes recorded during 30 days of storage at room temperature in response to adding BHT and BEO to sunflower oil.

PV is a measure of the degree of primary oxidation for oils and fats, indicating the occurrence of oxidation in the early stages [35]. During the 30 days of storage, a continuous increase in the PV value, proportional to the duration, was noticed in all the samples (*p* < 0.05) (Figure 1). The principal oxidation products, hydroperoxides, are responsible for the increase. The highest PV values were recorded in the case of the SFO control sample, within the range of 0.42–18.18 meq/kg oil. The PV decreased with the addition of BEO (0.42–16.24 meq/kg oil), the decrease was inversely proportional to the concentration of BEO added. The lowest values were recorded for the SFO + BHT sample, between 0.42–7.05 meq/kg.

#### 3.3.2. p-Anisidine Value (p-AV)

**Figure 2:**
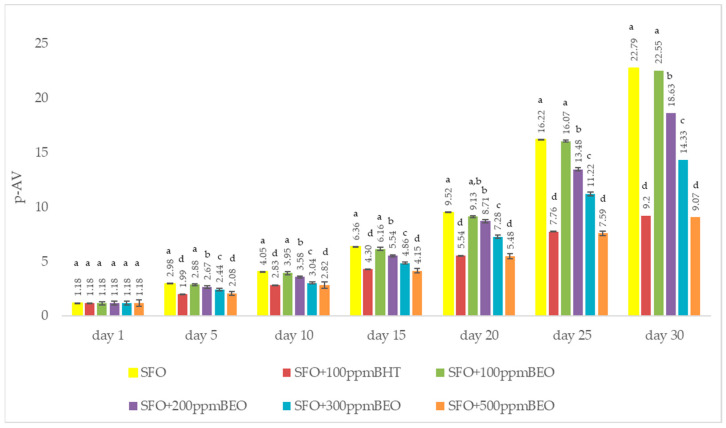
P-AV in oil samples as a response to BHT and BEO adding. The values are expressed as mean value ± standard deviations of three separate determinations. a–d ANOVA test was used to compare the average differences recorded between samples for the same day; mean values marked in different lowercase letters in ascending order show significant differences between the samples on the same day (*p* < 0.05); average values marked in identical lowercase letters show that there are no significant differences between samples on the same day (*p* < 0.05).

Figure 2 expresses the anisidine value recorded during the 30 days of storage in response to the addition of BHT and BEO.

The p-AV is a measurement of the secondary oxidation of lipids [35]. Analysing the results acquired for samples of sunflower oil containing BHT at varying concentrations of BEO, it was observed that with increasing storage time at room temperature, the p-AV value increases (*p* < 0.05) (Figure 2) due to the formation of secondary oxidation products.

The highest p-AV value was registered in the case of the control sample SFO, within the range of 0.18–22.79. The p-AV values decreased with the addition of BEO (1.18–22.55), decreasing as the concentration of BEO increased. The lowest values were recorded for the SFO + BHT sample, between 1.18 and 9.20.

Comparing the results obtained (*p* < 0.05) for the oil samples supplemented with BEO and the SFO + BHT sample, it was observed that the recorded values for SFO + 500 ppm BEO are close to those registered for SFO + BHT, even slightly lower, except for the ones remarked in the first and fifth day. A 500 ppm BEO concentration provided the greatest protection against the secondary oxidation of SFO. During the 30 days of storage at room temperature, a fact also evident in the peroxide value. Significant decreases in p-AV value were recorded in the case of SFO samples supplemented with BEO in the concentrations of 100 ppm, 200 ppm and 300 ppm compared to the SFO sample.

#### 3.3.3. Total Oxidation Value (TOTOX)

**Figure 3:**
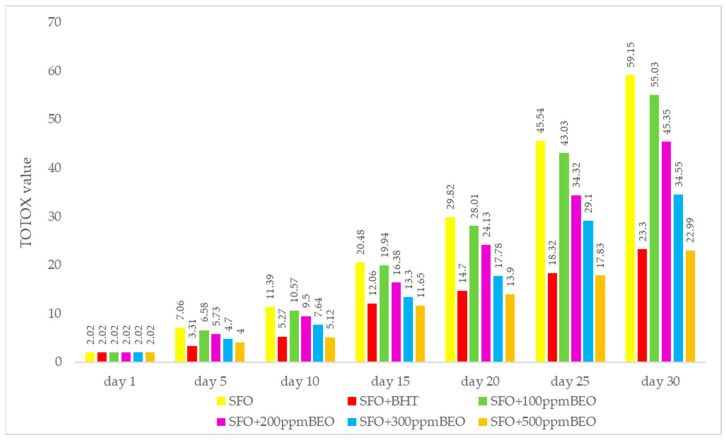
The impact of BEO and BHT on TOTOX value during the 30 days of storage.

PV provides information about the primary oxidation of the sample and p-AV about the secondary oxidation, but the two analyses together provide complete information about the entire oxidation process. The TOTOX value is a mathematical estimation of oxidative stability used worldwide to indicate oxidative stability in relation to the degree of oil degradation [20]. The TOTOX value of the analysed oil samples rose significantly as storage time increased. as seen in Figure 3.

Similar to PV and p-AV, in the case of TOTOX, the highest values were recorded for the control sample SFO throughout the 30 days of deposition, ranging between 2.02 and 59.15. TOTOX values decreased with the addition of BEO in SFO (2.02–55.03), decreasing as the concentration of BEO increased. The lowest values were recorded for the SFO + BHT sample, ranging between 2.02 and 23.3 (*p* < 0.05).

It can be noted that the values recorded for SFO + 500 ppm BEO are special those recorded for SFO + BHT, even slightly lower, except for the values recorded on day one and day 5. Significant decreases in p-AV value were also recorded in the case of SFO samples supplemented with BEO in concentrations of 100 ppm, 200 ppm and 300 ppm compared to the SFO sample.

#### 3.3.4. Assessing the Lipid Oxidation Degree of Oil Samples by Thiobarbituric Acid (TBA) Test

The TBA test is utilised to determine the level of secondary oxidation of vegetable oils, being one of the methods most often used in this regard. According to data from the specialised literature [36], after treatment with thiobarbituric acid, malondialdehyde can be dosed, leading to red condensation product formation, with absorption at 532–535 nm, the amount of malondialdehyde formed following the oxidation process can be quantified based on the calibration curve.

**Figure 4:**
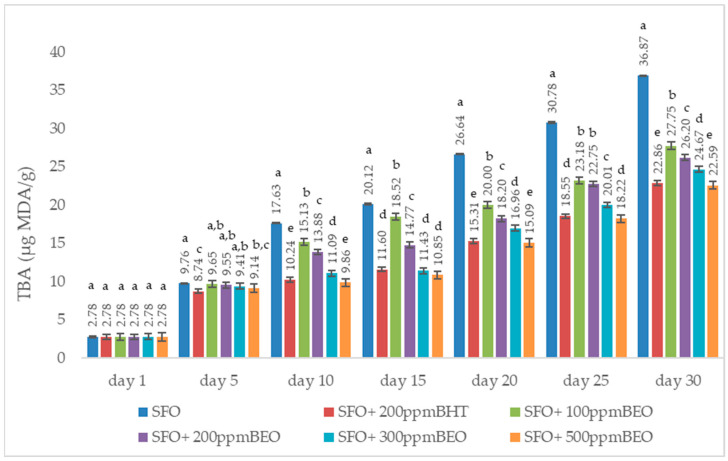
TBA value in oil samples in response to the addition of BHT and BEO. The values are expressed as the mean, standard deviation of three independent measurements. a–e ANOVA test was used to compare the average variances between samples collected on the same day; mean values marked in different lowercase letters in ascending order show significant differences between the samples on the same day (*p* < 0.05); average values marked in identical lowercase letters show that there are no significant differences between samples on the same day (*p* < 0.05).

The data shown in Figure 4 represent the changes in TBA values during the 30 days of storage due to the supplementation of sunflower oil with BHT and BEO (*p* < 0.05).

The evolution of the TBA values, recorded for the studied oil samples over a 30-day storage period, was followed. The highest values were recorded for the control sample (SFO), ranging between 2.78 and 36.87 µg MDA/g, significantly lower for the samples in which BHT or BEO was added. In the case of samples substituted with BEO, it can be observed that the values decreased with the increase in the added BEO concentration. In the case of the SFO + 500 ppm BEO sample, the values are even lower (2.78–22.59 µg MDA/g) than those recorded for the SFO + BHT sample (2.78–22.86 µg MDA/g). The *t*-test showed us that, except for day one and day 5, For each measurement period, there were statistically significant differences (*p* 0.05) between the TBA values of the control sample (SFO) and the samples supplemented with BHT and BEO (100, 200, 300 and 500 ppm, respectively). Additionally, potential differences between the TBA of BEO (100, 200, 300, and 500 ppm) and BHT values were investigated.

### 3.4. Anti-Inflammatory Activity

#### 3.4.1. Membrane Lysis Assay

The results obtained by membrane lysis assay are presented in Table 5.

As observed in Table 5, the inhibition of haemolysis started at a concentration of 4% of the *Boswellia* essential oil tested, with a percentage value of only 1.627. The highest concentration of *Boswellia* oil tested, at 16%, determined a percentage of inhibition of 26.25%, while dexamethasone protects the red cell from haemolysis in the proportion of 64.90%. The IC50 value obtained was 7.514%.

#### 3.4.2. The Effect on Protein Denaturation

The protective activity results against the protein’s thermal denaturation are presented in Table 6.

### 3.5. Antimicrobial Activity

The values of the OD for each tested strain, with different concentrations of *Boswellia* oil, are presented in Table 7.

**Figure 5:**
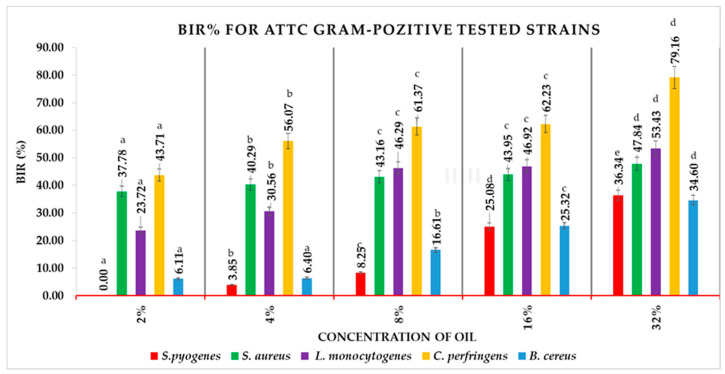
BIR% values of *Boswellia* oil on Gram-positive ATCC. Values are expressed as mean value ± standard deviations of three separate determinations. a–e ANOVA test was used to compare mean differences recorded between different concentrations of the same strain; mean values marked with different lowercase letters in ascending order indicate significant differences (*p* < 0.05); mean values marked with identical lowercase letters indicate no significant differences between different concentrations of the same strain (*p* < 0.05).

BIR% for all ATCC Gram-positive bacterial strains had positive values, different from one concentration to another of the tested *Boswellia* oil (Figure 5). The 2% concentration of the tested oil showed BIR% values between 0% for *S. pyogenes* and 37.78% for *S. aureus*. Similarly, the positive values of BIR% remain minimal for *S. pyogenes* (3.85%) and maximal for *S. aureus* (40.29%) at an oil concentration of 4%. Instead, the 8% oil concentrations cause a significant increase of BIR% for *L. monocytogenes*, reaching 46.29%, compared to the 30.56%, the value observed at a concentration of 4% of the oil. For the 8% concentration of the tested essential oil, the BIR% minimum value is noted for *S. pyogenes* (8.25%) and the maximal for *L. monocytogenes*. Similarly, for 16% and 32% concentrations, the BIR% knows the minimal value for *S. pyogenes* (22.08%, respectively 36.34%) and maximum for *L. monocytogenes* (46.92%, respectively 53.43%).

Depending on the essential oil concentrations, the BIR% for *S. aureus* had values between 37.78% and 47.84%, for *S. pyogenes* between 0.00% and 36.34% and for *L. monocytogenes* between 23.72% and 53.43%.

For *S. pyogenes*, the MIC was achieved at a concentration of 4%, all the subsequent values being positive, as presented in Table 8. All the other Gram-positive strains demonstrated a MIC value at the lowest concentration tested (2%), the trend being positive in all the cases.

**Figure 6:**
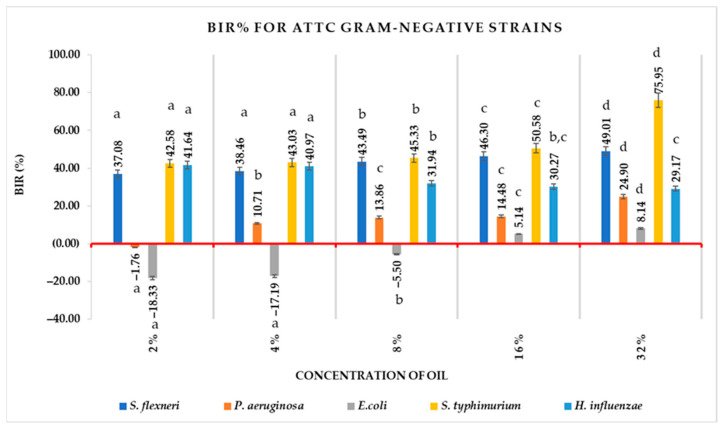
Antimicrobial activity (expressed as BIR%) of *Boswellia* oil on Gram-negative ATCC. Values are expressed as mean value ± standard deviations of three separate determinations. a–d ANOVA test was used to compare mean differences recorded between different concentrations of the same strain; mean values marked with different lowercase letters in ascending order indicate significant differences (*p* < 0.05); mean values marked with identical lowercase letters indicate no significant differences between different concentrations of the same strain (*p* < 0.05).

For Gram-negative ATCC strains, BIR% varied widely depending on the essential oil concentration and the studied strain, starting with positive values, except for *P. aeruginosa* and *E. coli*, from a concentration of 2% BEO, for most strains (Figure 6). The values of BIR% using a 2% concentration of BEO were positive for *S. flexneri* (37.08%), *S. typhimurium* (42.58%), *H. influenzae* (41.64%), *C. perfringens* (43.71%) and *B. cereus* (6.11%), and negative for *P. aeruginosa* (−1.76%) and *E. coli* (−18.33%). Unlike the 2% concentration of BEO, the 4% concentration determined the positivity of the BIR% value for *P. aeruginosa* (10.71%) but not for *E. coli* (−17.76%). Even at a concentration of 8% of BEA, the BIR% value for *E. coli* was negative, at −5.50% and became positive only when a concentration of 16% was used. Concentrations of 16, at 32% inhibited the growth of all Gram-negative ATCC strains. Except for *H. influenzae*, each strain had BIR% values inscribed into an ascending curve with a minimal value starting at a 2% concentration and maximal at 32%concentration of BEO. The BIR% curve of values for *H. influenzae* was descending, with maximum value observed at a 2% concentration and minimal at a 32% concentration of BEO.

Each ATCC Gram-negative strain had different BIR %values, depending on the BEO concentration. BIR% values for *S. flexneri* were between 37.08% at the lowest concentration studied of BEO and 49.01% at the highest studied concentration. For *S. typhimurium,* BIR% values were between 42.58% and 75.95%; *H. influenzae* between 41.64% and 29.17%; *C. perfringens* between 43.71 and 79.16%; *B. cereus* between 6.11% and 34.60%. For *P. aeruginosa* and *E.coli*, the BIR% values started from negative −1.75%, respectively −18.33%, reaching positive at the highest studied oil concentration (24.90% for *P. aeruginosa* and 8.14% for *E. coli*). Those being exposed showed that the MIC values for *S. flexneri*, *S. typhimurium*, *H. influenzae*, *C. perfringens* and *B. cereus* were 2%, while for *P. aeruginosa* and *E. coli* were 4%, respectively 16%.

In the case of the Gram-negative strains *Shigella*, *Salmonella* and *Haemophilus,* the MIC was found at the first concentration tested (2%).

**Figure 7:**
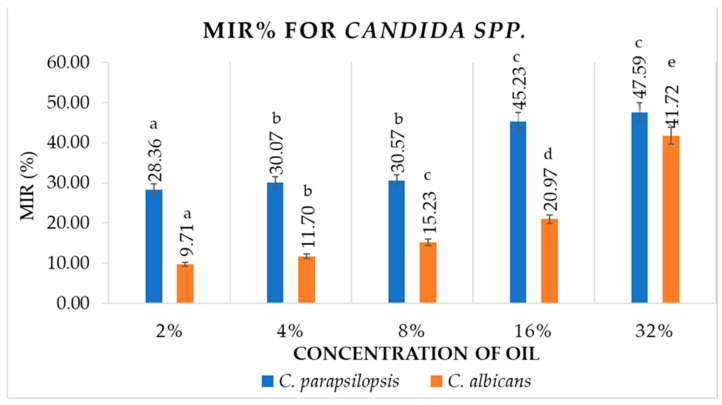
BIR% values of BEO on *Candida* ATCC strains. Values are expressed as mean value ± standard deviations of three separate determinations. The a–e ANOVA test was used to compare mean differences recorded between different concentrations of the same strain; mean values marked with different lowercase letters in ascending order indicate significant differences (*p* < 0.05); mean values marked with identical lowercase letters indicate no significant differences between different concentrations of the same strain (*p* < 0.05).

BEO has demonstrated antimycelial efficacy even at the lowest concentration studied, as presented in Figure 7. A concentration of 2% determined a value of BIR% for *C. parapsilopsis* ATCC strains of 28.36% and *C. albicans* of 9.71%. The 4%, 8%, 16% and 32% concentrations also inhibited mycelial growth, with positive values for BIR% increasing as the oil concentration increased.

The BIR values for each strain are inscribed in an ascending curve, with the maximum value at 32% concentration of BEO. So, the values of BIR% for *C. parapsilopsis* were between 28.36% and 47.59%, while for *C. albicans* were between 9.71 and 41.72%. As can be seen, in the case of mycelial ATCC strains, the increase in BEO concentration is directly proportional to the antifungal efficacy, at least up to a concentration of 32%.

Table 8 The MIC (%) for *Boswellia* oil on the tested ATCC strains. The samples that had no inhibition effect are marked in white. The light grey colour represents the samples where the MIC was found, but subsequent concentrations showed a potentiating effect. Therefore, the effect decreased together with the concentration. The red colour highlights the samples where the MIC was determined. The yellow colour is for the samples in which the effect was maintained together with increased concentration. The dark grey is for samples in which the MIC was not achieved, proving negative inhibitory values but positively correlated with the increase in concentration.

Table 9 represents the IC50 values calculated based on the OD values recorded for *Boswellia* oil on the tested ATCC strains.

IC50 showed higher values for the Gram-positive bacteria, ranging from 2.61% to 11.89% and for Gram-negative bacteria, from 1.70% to 8.68%. Concerning the sensibility, *C. albicans* proved to be the first affected, with an IC50 of 0.78%.

### 3.6. Molecular Docking Analysis

**Figure 8:**
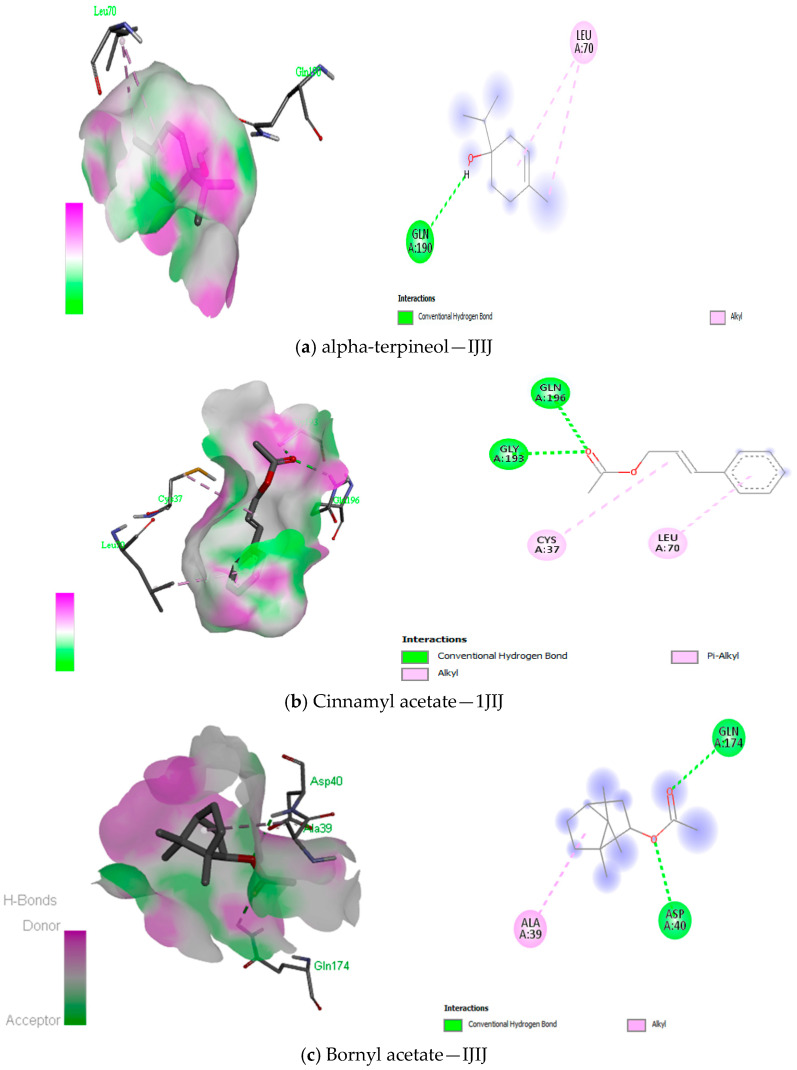

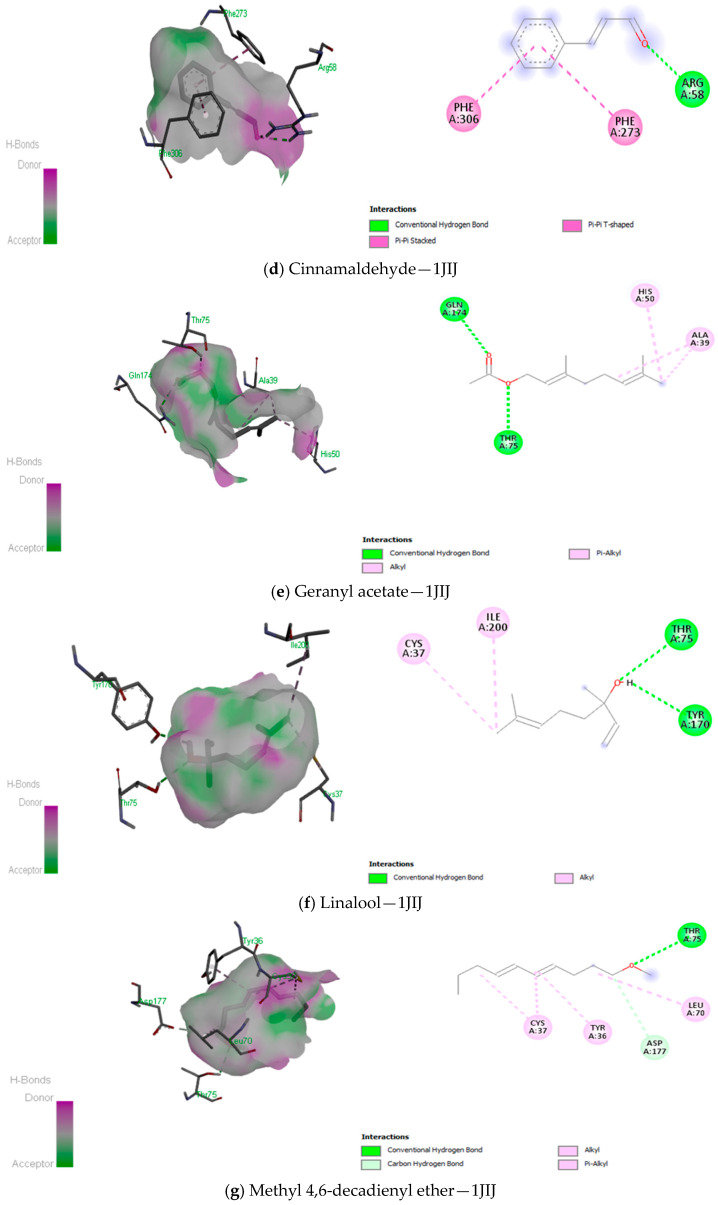

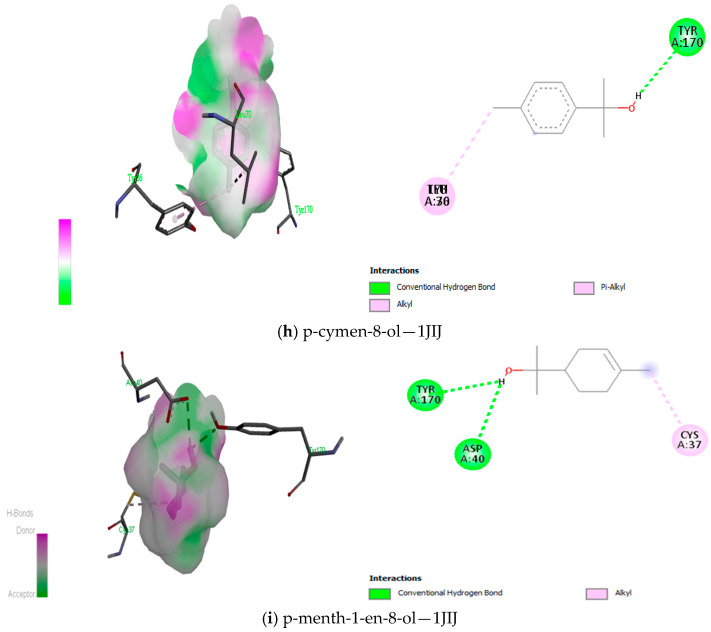
(**a**–**i**) 3D (three dimensions) and 2D (two dimensions) pictorial display of the most important interaction between the TyrRS amino acid residues and primary constituents of *Boswellia* oil.

**Figure 9:**
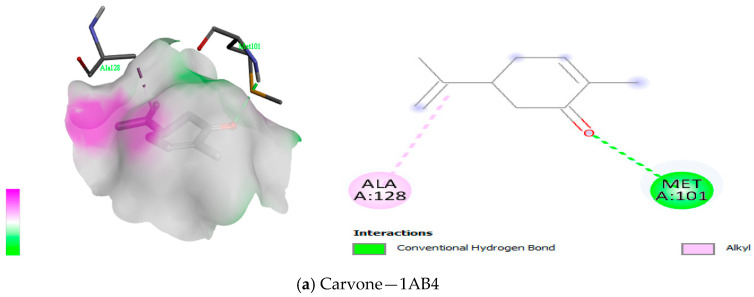

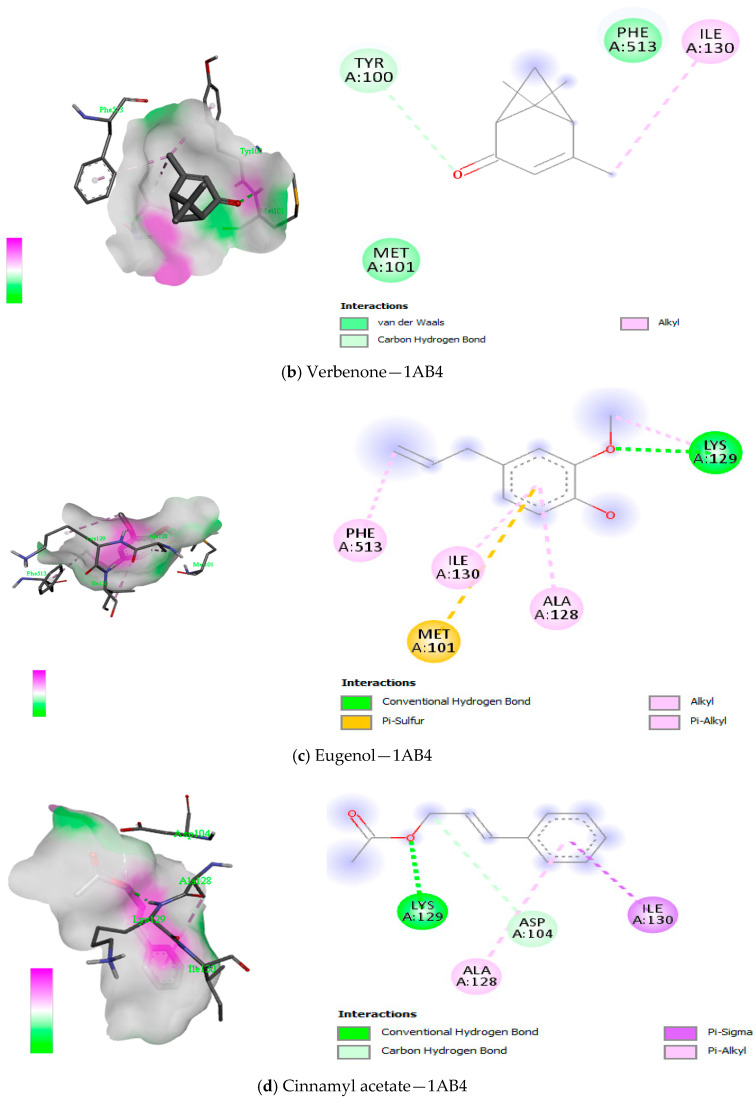

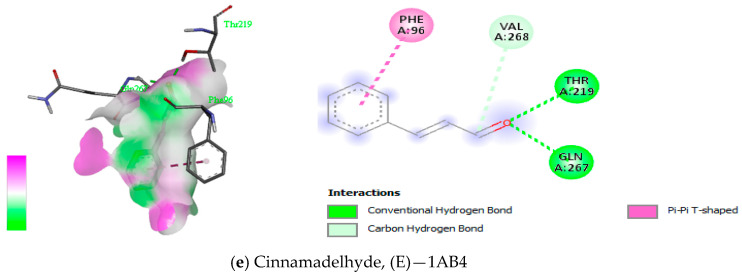
(**a**–**e**) 3D (three dimensions) and 2D (two dimensions) pictorial display of the most important interaction between the DNA gyrase amino acid residues and primary constituents of *Boswellia* oil.

**Figure 10:**
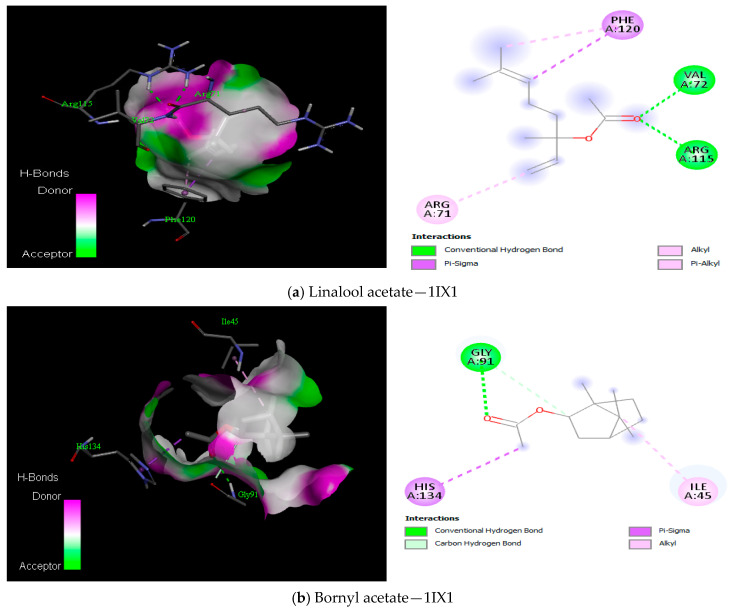

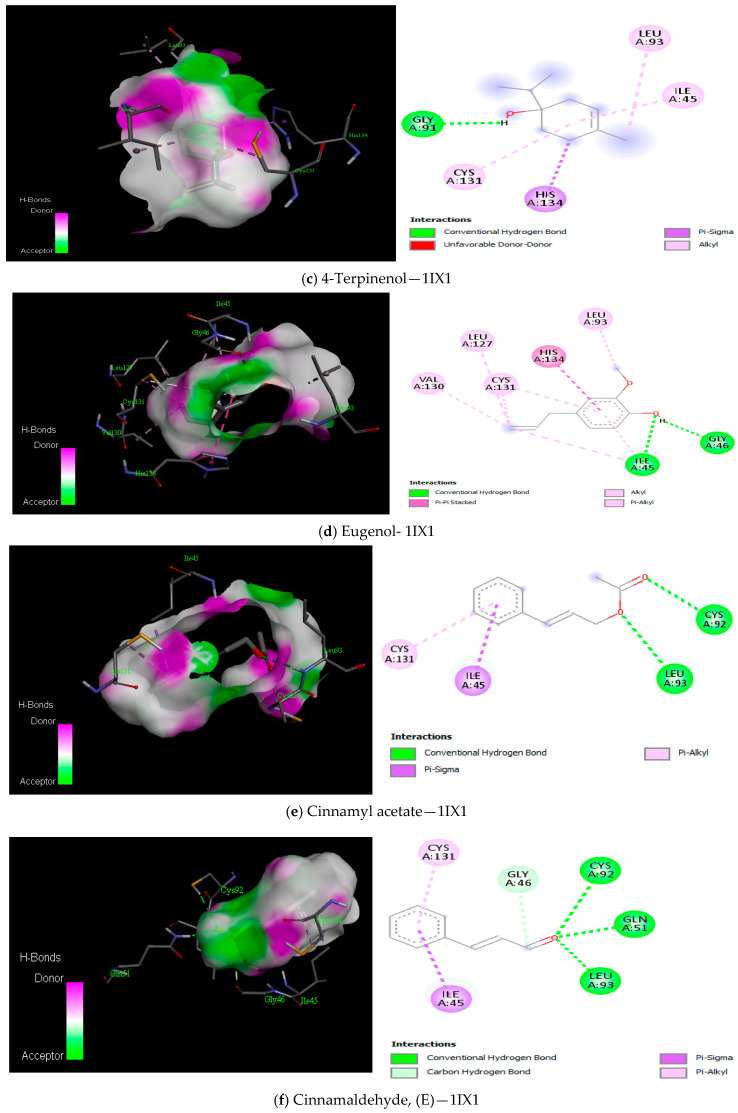
(**a**–**f**) 3D (three dimensions) and 2D (two dimensions) pictorial display of the most important interaction between the peptide deformylase amino acid residues and primary constituents of *Boswellia* oil.

**Figure 11:**
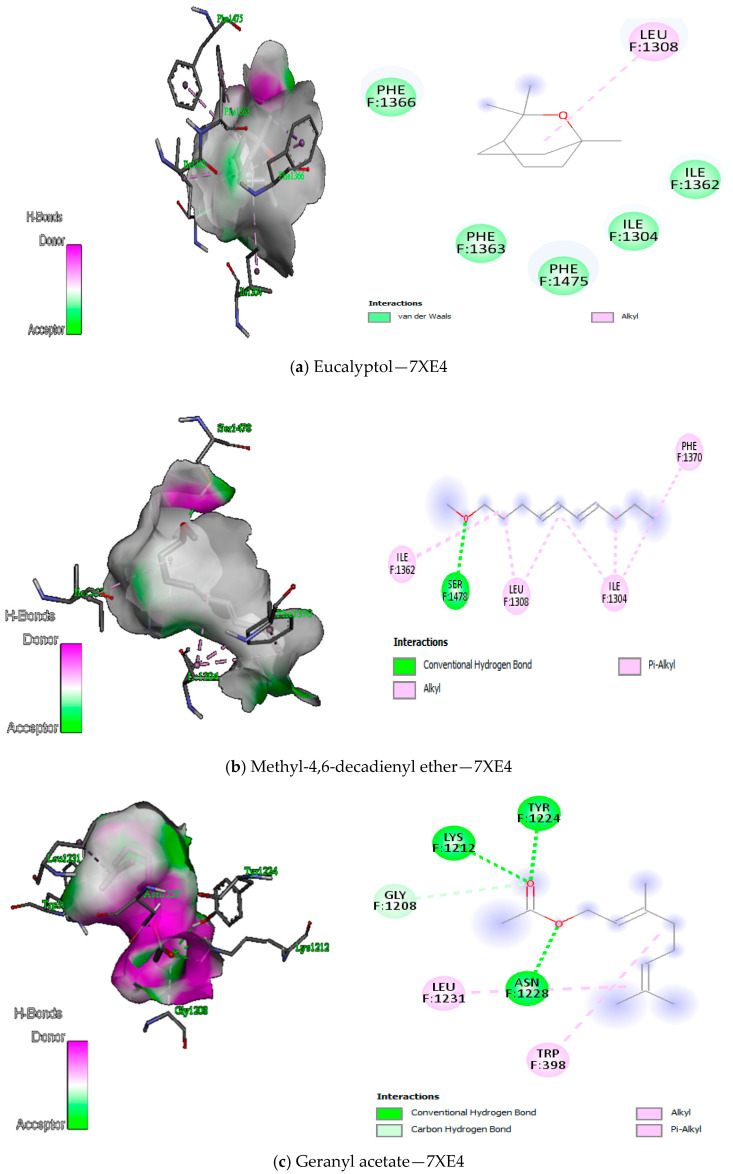

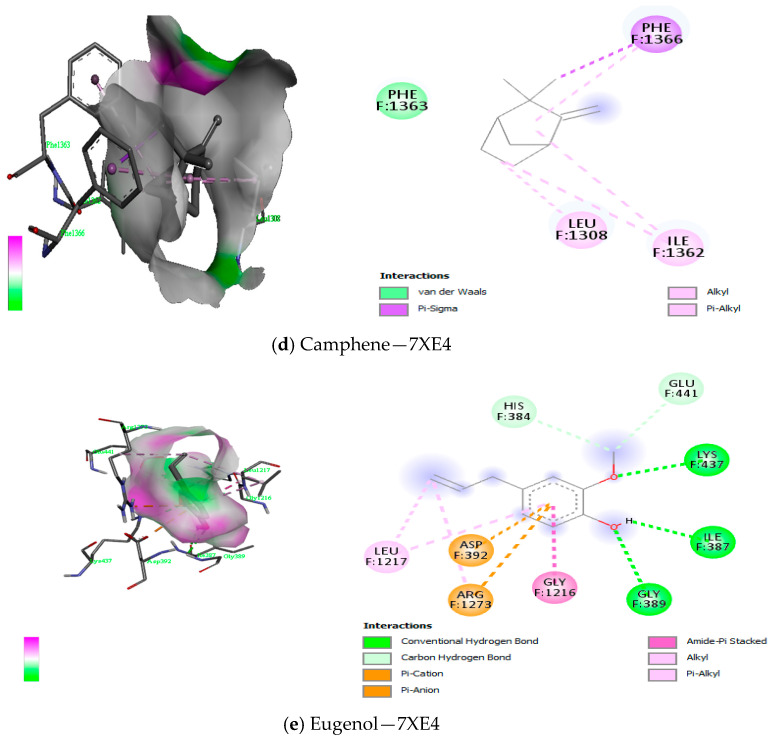
(**a**–**e**) 3D (three dimensions) and 2D (two dimensions) pictorial display of the most important interaction between the 7XE4 amino acid residues and primary constituents of *Boswellia* oil.

The docking of the compounds from *Boswellia* oil against TyrRS protein showed the lowest binding energies from −7.4 kcal/mol to −3.2 kcal/mol (Table 10), DNA gyrase protein showed binding energies from −7.1 kcal/mol to −4.4 kcal/mol (Table 11), peptide deformylase showed binding energies from −7.6 kcal/mol to −4.7 kcal/mol (Table 12). Both Methyl-4,6-decadienyl ether and geranyl acetate had the binding affinities of −5.1 kcal/mol and −5.8 kcal/mol to the protein, even though they had a GC-MS abundance of 0.24% and 0.36%, respectively. However, the visualisation of the receptor-ligand interaction demonstrated interesting H-bond interactions between the amino acid residues of the protein TyrRS and these two *Boswellia* oil compounds (Figure 8). We also observed that eugenol, cinnamyl acetate, and cinnamaldehyde had good binding interaction with DNA gyrase despite their low abundance, while linalool acetate, bornyl acetate, eugenol, cinnamyl acetate and cinnamaldehyde had the best binding interaction with peptide deformylase (Figure 9 and Figure 10). Altogether, the H-bonds were mostly between the compounds and the amino residues ARG58, ASP40, ASP177, CYS37, GLN174, GLN196, GLU 302, GLY38, GLY193, THR75, TYR170, among other C-H and alkyl bonds. Similarly, the docking of the compounds from *Boswellia* oil against 1,3-β-glucan synthase protein revealed the binding energies from −7.6 kcal/mol to −3.1 kcal/mol (Table 13). Importantly, eucalyptol, geranyl acetate, eugenol, and Methyl-4,6-decadienyl ether showed the best binding interaction with the protein (Figure 11). Although their abundance in the oil from the GC-MS is low, they were able to form a H-bond with the protein in a more interesting fashion. Thus, LYS437, ILE367, GLY389, ARG530, LYS1212, TRP1224, and ASN1228 were the amino acid residues of the protein that showed the H-bonds with these compounds amongst other C-H bonds and Alkyl/Pi-Alkyl bonds. Additionally, docking results of enantiomers of some of the compounds of *Boswellia* oil showed promising binding interaction with the four protein targets (Supplementary File S2, Tables S1–S4 and Figures S1–S4).

## 4. Discussion

### 4.1. Chemical Composition

The studies from the literature demonstrate differences in the chemical compositions of *Boswellia* oil, which are explained by the influence of geographical location and environmental conditions. Variation within the major constituents of the same type of oil suggests the possible existence of different chemotypes. Most *Boswellia carteri* and *Boswellia sacra* essential oils are dominated by α-pinene, followed by α-thujene, limonene, myrcene, sabinene, and p-cymene [3,37,38,39]. In contrast, another study found limonene and (E)-β-cymene the primary compounds in *B. sacra* oil [40]. α-pinene is the second most prevalent substance, according to Camarda et al. [41], who cited limonene as the most abundant component [41]. A recent study highlighted new chemical constituents in the *B. sacra* resins, β-boswellic aldehyde and 3β, 11β-dihydroxy BA, respectively, along with known α-amyrin (3-epi-α-amyrin, β-amyrin and α-amyrin) [42]. Regarding *B. frereana* essential oils, most studies demonstrated that the chemical composition contains, as a dominant constituent, α-pinene and lower levels of sabinene and p-cymene [39,43]. Another chemotype of *B. frereana* is dominated by α-thujene, the same constituent found at the highest level in *B. serrata* oil from India [44,45]. Evaluation of the chemical structure of the essential oil used in this study, a combination of *B. carteri*, *B. sacra*, *B. papryfera*, *B. frereana*, highlighted that the dominant compound was α-pinene, followed by limonene.

The chemical composition of different *Boswellia* oils is variable by the dominant constituent and the concentration of each compound in the same chemotype. Van Vuuren et al., 2010 [39] reported in different *Boswellia* oils a concentration of α-pinene (2.0–64.7%); myrcene (1.1–22.4%); sabinene (0.5–7.0%); β-caryophyllene (0.1–10.5%); limonene (1.3–20.4%); α-thujene (0.3–52.4%); p-cymene (2.7–16.9%); β-pinene (0.3–13.1%) and 10.5% β-caryophyllene. The same authors highlighted a percentage of α-pinene between 18.30–22.50% for *B. sacra* and 12.0–40.4% for *B. carteri* [39]. Grbic et al. [46] found a higher value of α-pinene (38.41%) in *B carteni* oil as a dominant compound, followed by myrcene (15.21%) [46]. In contrast, Di Stefano et al. [5] found the highest concentration of α-pinene in *B. sacra* essential oil, between 71.09–79.59%, depending on the geographical zone of the plant, followed by δ-3-carene (2.16–9.94%), camphene (3.00–3.23%) and β-pinene (2.17–2.39%). The same authors remarked that prolonged hydrodistillation could reduce the concentration of monoterpenes and increase the concentration of sesquiterpenes (β-elemene, β-eudesmene, γ-cadinene). However, the concentration of α-pinene remains at high values, not lower than 61.82% [5]. These concentrations of α-pinene, obtained by other authors, are higher than those observed in the present study, where although this component is dominant, it represents 39.34%. Moreover, the second noted chemical compound was limonene, with a proportion of 13.79%, followed by α-phellandrene (5.48%) and p-Cymene (4.19%). Close values of limonene to the present study were cited in the literature (18.20%) [41], while other studies reported values of the same compound almost double, mentioning that it was the dominant compound of *B. sacra* essential oil [40].

A comparative study of the commercial *Boswellia* oil from India with samples collected from Shivpuri Forest (northwestern district of Madhya Pradesh, India) demonstrated that the commercial samples contained a higher percentage of monoterpene hydrocarbons (81.9–88.1%), including α-thujene (61.4–69.8%) as the major compound. The wild samples are characterised by a higher percentage of oxygenated monoterpenoids/benzenoids (15.7%) and sesquiterpenes (19.2%), including α-terpineol (7.8%), terpinyl isobutyrate (5.1%), and eudesmol (11.5%) [44]. In contrast, the present study highlighted a low proportion of thujene in the commercial *Boswellia* oil, at only 3.00%, followed by isomenthone, cis- (2.86%), linalool acetate (2.58%), linalool (2.47%), menthyl acetate (2.03%) and caryophyllene (2.03%). Other compounds were found at a value under 2%. The diverse source plant can explain these differences regarding the chemical composition of the oil, different geographical areas and the extraction process, mentioning that the oil used in the present study contains several species of *Boswellia*.

### 4.2. Antioxidant Profile

The percentage of DPPH inhibition at 100 µg/mL was >80%, at 80 µg/mL >70%, at 70 µg/mL > 50%, at 60 µg/mL it was >20%, and at 50 µg/mL it was >10%.

As can be seen from the values shown in Table 3, the maximum radical scavenging activity was recorded for the highest concentration (100 µg/mL).

The percentage of DPPH inhibition remained high for the next two lower concentrations (80 µg/mL and 70 µg/mL, respectively), comparable to those recorded for ascorbic acid. At the lowest concentration tested (50 mg/mL), the antioxidant activity showed a significant decrease, the values being comparable to that recorded for ascorbic acid.

Similar to the present study, Ayub et al. found a value of AA between 56.74 ± 0.79 and 94.39 ± 1.04% for the essential oils of *Boswellia serrata* [47]. The AA values obtained are consistent with the values reported by isolated by different extraction methods, respectively. Another study conducted by Mothana et al. demonstrated weaker antioxidant abilities of three *Boswellia* essential oils (*B. dioscorides*, *B. elongate* and B. *socotrana*) in reducing DPPH (22%, 21%, and 28%) at a concentration of 1 mg/mL [48].

The IC50 values (Table 3) were 249.37 µg/mL for BEO and 228.40 µg/mL for the ascorbic acid control sample.

Similar results in terms of antioxidant activity have been recorded in other studies conducted for *Boswellia* essential oil. Thus, Ali et al. [49] reported for *Boswellia socotrana* an IC50 value of 121.4 μg/mL, for *B. elongata* 211.2 μg/mL) and for *B. ameero* 175.2 μg/mL. In the same study, an inhibitory activity of 59.3% for oils obtained from *B. socotrana* at a concentration of 200 μg/mL compared to *Boswellia elongata* and *Boswellia ameero* essential oil, for which inhibition of 29.6% and 41.6%, respectively, was reported [49]. Mothana et al. [48] reported for essential oils obtained from *Boswellia* species (*B. dioscorides*, *B. elongata* and *B. socotrana*) weak antioxidant activities (28%) at 1.0 mg/mL. Kohoude et al. [50] studied the chemical composition and biological activity of extracts and essential oil of *Boswellia dalzieliileaves*, reporting an inhibition of 11.54 ± 0.20% at a concentration of 100 mg/L against DPPH radicals.

### 4.3. Oxidative Stability

#### 4.3.1. Peroxide Value

Comparing the results obtained for the oil samples supplemented with BEO versus the SFO + BHT sample, it was observed that, except for the values recorded on the first and fifth day, the values recorded for SFO + 300 ppm BEO are close to those registered for SFO + BHT. The SFO + 500 ppm BEO values are significantly lower than those recorded for SFO + BHT. So, a concentration between 300–500 ppm BEO can successfully replace the synthetic antioxidant BHT.

Cocan et al. studied the antioxidant effect of hot pepper and sweet pepper seed oil for stabilising sunflower oil and obtained similar results to the present study [23]. Jianu et al. [51] investigated the effectiveness of *Mentha* × *smithiana* R. graham essential oil compared with butylhydroxytoluene (BHT) on the delay of lipid oxidation of sunflower oil during 24 days of room temp. The author reported that supplementing cold-pressed sunflower oil with various concentrations of *Mentha* × *smithiana* R. graham essential oil can inhibit the process of lipid oxidation [51]. In another study, Tena et al. reported the stabilising effect of spearmint or pomegranate essential oils on sunflowers [52].

#### 4.3.2. p-Anisidine Value (p-AV)

The results are consistent with the values reported by other authors for strengthening the oxidative stability of sunflower oil by supplementing it with other oils. Cocan et al. studied the effect of hot pepper and sweet pepper seed oil in stabilising sunflower oil [23]. Alsufiani and Ashour also compared 2,4,4′-Trihydroxychalcone as a natural antioxidant to butylhydroxytoluene (BHT) on the delay of lipid oxidation of sunflower oil during 88 days of rest at room temperature [53]. In another study, Wang et al., at a concentration of 800 ppm, the antioxidant effect of the essential oil of *Punica granatum* cv. *Heyyinshiliu* in stabilising sunflower oil was reported, with encouraging results [54].

#### 4.3.3. Total Oxidation Value (TOTOX)

As with PV and p-AV, 500 ppm BEO provided the greatest protection against the secondary oxidation of SFO during 30 days of storage at room temperature. The results follow a similar trend to those reported by other authors for sunflower oil supplemented with other oils or extracts with protective activity [55,56].

#### 4.3.4. Assessing the Lipid Oxidation Degree of Oil Samples by Thiobarbituric Acid (TBA) Test

Throughout the 30 days of storage, close values were recorded for SFO + 500 ppm BOT and SFO + 200 ppm BHT (4.937 ± 0.195 µg MDA/g), indicating that BEO at a concentration of 500 ppm can replace the synthetic antioxidant. A similar trend was found by Hashemi et al. [56], who studied the effect of *Carum copticum* essential oil in different concentrations (0.025%, 0.05% and 0.075%) on the oxidative stability of sunflower oil compared to butylated hydroxyanisole (BHA) and butylated hydroxytoluene (BHT) during storage at 37 and 47 °C. When examining the impact of *Mentha × Smithiana* essential oil on sunflower oil, the author observed the same trend in TBA values. Okhli et al. also reported the same trend in their study on the effect of essential oil from the lemon peel (*Citrus medica* L.) on the stabilisation of sunflower oil [57]. In another study, Al-Dalain et al. studied the effect of essential oils extracted from fennel, rosemary and ginger on the oxidative stability of sunflower oil during storage at ambient temperature with exposure to light [58]. The obtained results showed that the studied essential oils inhibited the formation of primary and secondary oxidation products during the heating and storage of sunflower oil.

### 4.4. Anti-Inflammatory Activity

For centuries, gum-resin extracts of *Boswellia serrata* have been used in folk medicine to treat chronic inflammatory diseases [59]. In laboratory conditions, the anti-inflammatory activity of *Boswellia* extracts was highlighted by various methods such as membrane stability test, inhibition of albumin denaturation, inhibitory proteinase activity, and reduction in TNF-α, IL 1-β [60,61,62]. Some of the researchers demonstrated that *B. serrata* extracts are capable of antagonising the inflammatory effect of LPS in human and mouse macrophages, and monocytes [60,61,62,63], but also in endothelial cells [64]. The principles of these methods vary from one method to another, but their approach represents a primary stage in evaluating the anti-inflammatory effect.

Lysosomal membrane stabilisation is vital in controlling the inflammatory response by inhibiting the release of lysosomal constituents of activated neutrophils. The human red blood cell membrane is analogous to the lysosomal membrane, and its stabilisation implies that the extract may also stabilise lysosomal membranes [24,25]. In the present study, the maximum inhibitory haemolysis value (26.253%) was observed at the highest concentration of *Boswellia* essential oil tested, 160 µL/mL. At this concentration, the inhibitory value was slightly below half the value obtained by using dexamethasone at a concentration of 0.1 mg/mL. However, the protective activity of the red cell started from the concentration of 40 µL/mL, with a value of inhibition of 1.627%. In contrast, Gokulan et al. demonstrated the anti-inflammatory activity of an alcoholic extract of *Boswellia serrata* started from 100 µg/mL, with a value of inhibition of 15.6%, while the maximum concentration tested, 500 µg/mL, determined a percentage of inhibition of 65.62%, close to the value obtained by using 100 µg/mL of aspirin [65].

In inflammatory and arthritic diseases, denaturation of a protein is a process characterised by the loss of the protein’s biological functions [66]. The present study demonstrated that the protective activity against protein denaturation started at a concentration of 80 µL/mL, the percentage of inhibition being 15.024%. A 160 µL/mL concentration of BEO determined a protein denaturation percentage value of 25.055%. In contrast, Gokulan et al. found that the minimal inhibition concentration of *Boswellia serrata* was 100 µg/mL with a value of inhibition of 31.42% [65]. The increasing concentration determines the increase in the protective activity against protein denaturation, so the percentage of inhibition becomes 58.22 ± 3.84% by using 500 µg/mL of *Boswellia serratta* [65,67]. Moreover, the effectiveness of *Boswellia* extract as a potential anti-inflammatory drug for osteoarthritis has been also demonstrated in a lot of clinical trials [68,69,70], not only by using in vitro studies. It seems that *Boswellia* and its extract may relieve the pain and stiffness and improve the joint’s function when is administrated at least for 4 weeks [70]. Other clinical studies demonstrated that *B. serrata* used in combination with other herbs such as *Kaempferia galanga* [71] and *Curcuma longa* [72] had effects in relieving the symptoms of osteoartitis.

Another in vivo study demonstrating *Boswellia*’s anti-inflammatory effects was conducted on rats using the paw oedema method induced by carrageenan. According to Al-Harrasi et al., 2013 [73], *Boswellia sacra* oil inhibited the formation of oedema by 21.3%, 18.8%, 17.1%, and 25.8% after 1, 2, 3, and 4 h, the results being higher than aspirin [73].

Studies have demonstrated that the anti-inflammatory activity is attributed to the boswellic acids [2], so the variation in the chemical composition of the plant is attributed to this property. By extension, the results of the anti-inflammatory activity of the tested essential oil from the present study are encouraging, with further in vitro and in vivo studies being necessary for future practical applicability.

### 4.5. Antimicrobial Activity

Several studies described the antimicrobial efficacy of *Boswellia* essential oil [3,5,74,75] and highlighted different sensitivity from one bacterial strain to another. Comparing the antimicrobial efficacy against the two bacterial groups, studies indicate *Boswellia* volatile oils were more active against Gram-positive than Gram-negative bacteria [76]. According to Raja et al. [77], the lack of antibacterial activity of active compounds of *Boswellia* sp. against Gram-negative bacteria might be attributed to the outer membrane of these bacteria. Being composed primarily of lipopolysaccharide molecules, this membrane forms a hydrophilic permeability barrier that protects against the effects of highly hydrophobic compounds, respectively, against the acetyl-keto-β-*boswellia* acid (AKBA) [77]. Still, except for *P. aeruginosa* and *E. coli*, the present study demonstrates that Gram-positive strains have the same MIC as Gram-negative ones, respectively 2%, with an inhibition rate of around 40% both for *S. aureus* and *S. flexneri*, *S. typhimurium*, *H. influenzae*. *C. perfringens*, *B. cereus*. Moreover, the bacterial inhibition capacity of the BEO for *S. pyogenes* strains is evident at a concentration of 4% (40 mg/mL), the same MIC as for *P. aeruginosa*. Of all the strains studied, *E. coli* was the most resistant to BEO, with the highest MIC value at 8% (80 mg/mL). In contrast, Ayub et al. demonstrated that of all the studied strains, *E. coli* was the most sensitive to the *Boswellia* oil, presenting larger inhibition zones (7.57 ± 0.19–16.80 ± 0.33 mm) and smaller MIC values (70.36 ± 1.82–337.78 ± 4.52 µg/mL) [47]. However, Van Vuuren et al. [39] sustained that antimicrobial activity against *E. coli* varied between 4.0–12.8 mg/mL, with a mean average of 6.2 ± 1.8 mg/mL depending on the type of *Boswellia* oil sample. The same author demonstrated that *B. cereus* exhibited the most noteworthy antimicrobial activity with MIC values ≤ 2 mg/mL [39]. Still, in the present study, the MIC of 2% (20 mg/mL) for *B. cereus* was similar to some Gram-negative or Gram-positive bacteria. A concentration of 2% (20 mg/mL) was active against *S. aureus*, *L. monocytogenes* as representatives of the Gram-positive class, and some strains of the Gram-negative group but not against *P. aeruginosa*. The observation contrasts with the data from the literature. Di Stefano et al., 2020, sustained that the MIC value against *S. aureus* and *P. aeruginosa* of Grade 2 essential oil of Najdi was 52 mg/mL. For the same strains, the MIC values of Grade 2 Sahli essential oil were higher, ranging from 440 to 110 mg/mL [5], which proves that the antimicrobial activity varied depending on the origin of the plant. However, Ayub et al., demonstrated that *S. aureus* was the least sensitive bacterial strain, with MIC values ranging from 98.52 ± 1.96–168.88 ± 1.96 µg/mL [47].

The study of fungal strains demonstrated that BEO is effective against *C. albicans* and *C. parapsilopsis* at 2% (20mg/mL). Di Stefano et al. [5] discovered that BEO exhibited antifungal activity against *C. albicans* and *M. furfur*, with MIC values ranging from 54.56 to 0.240 mg/mL. In particular, Grade 2 essential oil from Najdi and Grade 1 essential oil from Sahli showed MIC values at the lowest tested concentration, corresponding to a percentage *v/v* of 0.03 (≤0.252 mg/mL). In contrast, another study demonstrated moderate to poor activity of *Boswellia* oil, with MIC values ranging between 5.3–12.0 mg/mL, with a mean average of 7.4 ± 1.9 mg/mL [39].

The values of the antioxidant activity of *Boswellia* species natural products vary depending on plant species origin and extract type. Expressed by IC50, the antioxidant activity of the methanolic extracts from *Boswellia* serrata collected from different territories in India was demonstrated to be 2.7–9.9 µg/mL [78]. Essential oil of *B. dalzielii* leaves showed an IC50  =  6.10 mg/L [50], while for B. carteri was recorded at 0.64 μL/mL [79].

All these variations regarding the different MIC values of *Boswellia* oil against microbial strains can be justified by the different chemical composition of each oil, depending on climate, the geographical location of the plant source, the age of the tree, harvest season and last but not least, the processing method for obtaining it.

### 4.6. Molecular Docking

The current study analysed BEO antimicrobial potential on Gram-positive and Gram-negative bacteria strains. To further understand how the oil is potentiating its effects on these microorganisms, computational tools were employed to describe the mode of interaction between the bioactive compounds of the essential oil with the prokaryotic protein, TyrRS, DNA gyrase, peptide deformylase and 1,3-β-glucan synthase. The findings revealed interesting binding energies with compounds having low abundance (<1%). Table 10, Table 11, Table 12 and Table 13 all showed compounds having the lowest binding energies that interact better with all the proteins: cinnamyl acetate, bornyl acetate, cinnamaldehyde, linalool, p-cymen-8-ol, p-menth-1-3n-8-ol, eucalyptol and eugenol. The interactions of menthyl-4,6-decadienyl ether and geranyl acetate with TyrRS predicts hydrogen bonds with amino acid residues THR75 and GLN174 while having ASP177 forming a carbon-hydrogen bond and an alkyl bond, while LEU70, TYR36, CYS37, ALA39 and HIS50 formed pi-alkyl bond with the compound (Figure 8). Suffice to say that these compounds interacted with amino acids that are either polar, aromatic or sulfur-containing. Importantly, these amino acids were reportedly present in the active site of the TyrRS protein [9], as well as been stable in the active sites by strong hydrogen and other hydrophobic bonds for DNA gyrase and peptide deformylase (Figure 9 and Figure 10).

Meanwhile, eugenol also had the best interaction with 1,3-β-glucan synthase, wherein three hydrogen bonds were observed with LYS437, ILE387, GLY389 and a couple of carbon-hydrogen bonds with HIS384, GLU441, amidst other alkyl/pi-alkyl bonds (Figure 11e). Perhaps the free hydroxyl group in eugenol could be responsible for this strong interaction and ultimately add to the antimicrobial activity of the *Boswellia* essential oil [80]. Additionally, compounds with the highest abundance could not interact nicely with the protein, perhaps due to the restriction of the docking parameters to the active site of TyrRS, DNA gyrase and peptide deformylase. Seemingly, the same deduction was made between the compounds and the fungi 1,3-β-glucan synthase. However, previous reports demonstrated that compounds with low abundance in essential oils could render the desired bioactive properties to the overall function of the oil [81,82]. Similarly, physical factors such as storage and temperature may contribute to the compound’s low abundance of essential oil [83]. Thus, the presence of these compounds in the essential oils, however, their abundance would equally be bioactive against a wide range of bacteria and fungi target.

## 5. Conclusions

The present research characterised BEO regarding chemical composition, antioxidant, anti-inflammatory and antimicrobial activity. The chemical characterisation of BEO identified 36 compounds, and the major components were represented by α-pinene (39.34%) and limonene (13.79%). Evaluation of the antioxidant activity using the DPPH method showed high inhibition comparable to the synthetic antioxidant used as a control. Oxidative stability evaluation showed that BEO has the potential to inhibit primary and secondary oxidation products with almost the same efficacy as BHA. BEO at a concentration of 500 ppm provided the best protection against secondary oxidation during 30 days of storage at room temperature, which was also evident in the peroxide value. Concerning the anti-inflammatory activity, even if the value of protection was lower than the value registered in the case of dexamethasone, the recommendation of using BEO as a protective agent stands considering the lower side effects. Nevertheless, further in vitro and in vivo studies are necessary for future practical applicability. Regarding the antimicrobial activity, BEO proved more effective against Gram-positive bacteria and had almost no effect on *E. coli*. The computational tools employed to describe the mode of interaction between the bioactive compounds of the essential oil with the TyrRS, DNA gyrase, and peptide deformylase proteins revealed interesting binding energies with compounds having a low abundance, thereby supporting the antimicrobial and anti-inflammatory activities of the oil. Similarly, eugenol interactions had the best binding interaction with 1,3-β-glucan synthase. Even so, compounds with low abundance in BEO could render the desired bioactive properties to the overall function of the oil sustained by physical factors such as storage and temperature. Consequently, even if the docking results suggest a strong interaction between some compounds and the tyrosyl-tRNA synthetase, this has not been demonstrated in cells or in vitro; further analysis is being considered for future research.

## Figures and Tables

**Table 1:** Chemical composition of the tested frankincense essential oil detected by GC-MS.

No.	Chemical Compound	Percentage of Total Compounds (%) ^a^	Type of Compounds	LRI Reported in Literature [33]	LRI Determined Experimental
1.	alpha-Pinene	39.34 ± 0.082	MH	1001	1021
2.	Thujene	3.00 ± 0.018	MH	1040	1056
3.	beta-Pinene	1.89 ± 0.011	MH	1071	1106
4.	3-Carene	0.67 ± 0.001	MH	1138	1110
5.	alpha-Phellandrene	5.48 ± 0.021	MH	1154	1156
6.	beta-Myrcene	1.74 ± 0.010	MH	1149	1164
7.	Limonene	13.79 ± 0.027	MH	1164	1196
8.	Eucalyptol	1.21 ± 0.048	MO	1218	1204
9.	*p*-Cymene	4.21 ± 0.023	MH	1258	1241
10.	Acetic acid, octyl ester	3.71 ± 0.019	-	1464	1465
11.	τ-Elemene	0.51 ± 0.003	SH	1482	1484
12.	Linalool	2.47 ± 0.014	MO	1533	1532
13.	Linalool acetate	2.58 ± 0.005	MO	1545	1550
14.	Menthyl acetate	2.03 ± 0.012	MO	1539	1552
15.	Isomenthone	2.86 ± 0.015	MO	1555	1556
16.	Bornyl acetate	0.75 ± 0.011	MO	1562	1560
17.	Menthone	0.73 ± 0.001	MO	1568	1570
18.	4-Terpineol	0.97 ± 0.002	MO	1571	1573
19.	Verbenone	0.51 ± 0.001	MO	1580	1581
21.	Caryophyllene	2.03 ± 0.001	SH	1581	1598
22.	β-Elemene	0.50 ± 0.001	SH	1582	1599
23.	Caryophyllene oxide	2.26 ± 0.013	SO	1954	1960
24.	Eugenol	0.98 ± 0.004	PHT	2141	2192
	Total of major compounds	95.04%			
	Monoterpene hidrocarbonates (MH)	70.98%			
	Monoterpene oxygenate (MO)	14.09%			
	Sesquiterpene hidrocarbonates (SH)	3.03%			
	Sesquiterpene oxygenate (SO)	2.26%			
	Phenolic monoterpenoid (PHT)	0.98%			

^a^ compounds detected in percentages higher or equal as 0.5%.

**Table 2:** DPPH free radical scavenging of BEO.

Sample	AA (%)
BEO	86.44 ± 2.12 ^a^
BHT	89.06 ± 1.88 ^a^

The values are expressed as the mean standard deviations of three independent determinations. ^a^ The mean differences between BEO and BHT were compared using a *t*-test; values within the same superscripts are not statistically different (*p* > 0.05).

**Table 3:** The DPPH radical scavenging activity (% inhibition) of ethanolic extracts vs. ascorbic acid.

Concentration (µg/mL)	BEO	Ascorbic Acid
	Inhibition (%)	Inhibition (%)
50	15.18	25.22
60	29.45	54.48
70	52.24	65.24
80	70.25	82.32
100	86.44	94.54

**Table 4:** The IC50 value of BEO sample vs. ascorbic acid.

Samples	BEO	Ascorbic Acid
IC50 (µg/mL)	249.37	228.40
R^2^	0.9957	0.9913
Hill Slope	8.332	17.548

**Table 5:** The anti-inflammatory values obtained by membrane lysis assay.

The Concentrations of *Boswellia* Oil	The OD Values	% of Haemolysis	% Inhibition of the Haemolysis
10 µL/mL	2.947 ± 0.001	117.745	-
20 µL/mL	2.803 ± 0.005	112.008	-
40 µL/mL	2.462 ± 0.005	98.373	1.627
80 µL/mL	1.996 ± 0.008	79.765	20.235
160 µL/mL	1.846 ± 0.002	73.747	26.253
Control sample with dexamethasone	0.878 ± 0.003	35.100	64.900
Control sample with PBS	2.503 ± 0.005	100	-

**Table 6:** The effect of protein denaturation obtained by using different concentrations of BEO.

The Concentrations of *Boswellia* Oil (%)	The OD Values	% of Protein Denaturation	% Inhibition of Protein Denaturation
10 µL/mL	1.326 ± 0.003	100.990	-
20 µL/mL	1.321 ± 0.003	100.630	-
40 µL/mL	1.317 ± 0.002	100.307	-
80 µL/mL	1.116 ± 0.003	84.976	15.024
160 µL/mL	0.984 ± 0.004	74.945	25.055
Control sample with dexamethasone	0.472 ± 0.003	35.925	64.075
Control sample with PBS	1.313 ± 0.004	100	-

**Table 7:** The OD reading of the different concentrations of *Boswellia* oil against the tested strains.

The Concentration of Oil	The ATCC-Tested Strains											
	*S. aureus* (ATCC 25923)	*S. pyogenes* (ATCC 19615)	*L. monocytogenes* (ATCC 19114)	*P. aeruginosa* (ATCC 27853)	*E. coli* (ATCC 25922)	*S. typhimurium* (ATCC 14028)	*S. flexneri* (ATCC 12022)	*H. influenzae* (ATCC 10211)	*B. cereus* (ATCC 10876)	*C. perfringens* (ATCC 13124)	*C. parapsilosus* (ATCC 22019)	*C. albicans* (ATCC 10231)
2%	0.447 ± 0.002	0.598 ± 0.005	0.926 ± 0.005	0.712 ± 0.002	0.867 ± 0.004	0.641 ± 0.002	0.425 ± 0.003	0.616 ± 0.004	0.543 ± 0.004	0.436 ± 0.003	0.476 ± 0.004	0.136 ± 0.005
4%	0.429 ± 0.005	0.575 ± 0.003	0.843 ± 0.002	0.625 ± 0.005	0.859 ± 0.004	0.636 ± 0.003	0.416 ± 0.002	0.623 ± 0.002	0.541 ± 0.003	0.340 ± 0.001	0.464 ± 0.005	0.133 ± 0.002
8%	0.409 ± 0.004	0.549 ± 0.005	0.652 ± 0.003	0.603 ± 0.004	0.773 ± 0.004	0.611 ± 0.001	0.382 ± 0.002	0.719 ± 0.003	0.482 ± 0.003	0.299 ± 0.002	0.461 ± 0.004	0.128 ± 0.001
16%	0.403 ± 0.004	0.448 ± 0.005	0.644 ± 0.002	0.599 ± 0.002	0.695 ± 0.003	0.552 ± 0.007	0.363 ± 0.004	0.736 ± 0.004	0.432 ± 0.002	0.292 ± 0.002	0.364 ± 0.003	0.119 ± 0.002
32%	0.375 ± 0.003	0.381 ± 0.001	0.565 ± 0.003	0.526 ± 0.004	0.673 ± 0.003	0.269 ± 0.003	0.345 ± 0.004	0.748 ± 0.003	0.378 ± 0.003	0.161 ± 0.003	0.348 ± 0.004	0.088 ± 0.002
BHI	0.719 ± 0.004	0.598 ± 0.017	1.214 ± 0.003	0.700 ± 0.003	0.733 ± 0.003	1.117 ± 0.004	0.676 ± 0.003	1.056 ± 0.007	0.578 ± 0.004	0.774 ± 0.004	0.664 ± 0.004	0.151 ± 0.004

**Table 8:** The MIC (%) for *Boswellia* oil on the tested ATCC strains.

	2%	4%	8%	16%	32%
*S.pyogenes*					
*S. aureus*					
*L. monocytogenes*					
*Cl. perfringens*					
*B. cereus*					
*S. flexneri*					
*P. aeruginosa*					
*E.coli*					
*S. typhimurium*					
*H. influenzae*					
*C. parapsilopsis*					
*C. albicans*					

**Table 9:** IC50 values calculated based on the OD values.

ATCC Strains	IC50 (%)
*S.pyogenes*	11.89
*S. aureus*	3.731
*L. monocytogenes*	2.61
*Cl. perfringens*	6.00
*B. cereus*	5.84
*S. flexneri*	1.70
*P. aeruginosa*	6.79
*E. coli*	8.68
*S. typhimurium*	3.83
*H. influenzae*	2.15
*C. parapsilopsis*	1.69
*C. albicans*	0.78

**Table 10:** Binding energies and bonding interaction between the chemical compounds of *Boswellia* oil and TyrRS protein (PDB:1JIJ).

S/No	Chemical Compound of the Frankincense Oil	Binding Energies (kcal/mol)	Bonding Type (TyrRS + Compound)
1	.alpha.-Pinene	−5.6	Pi-Sigma/Pi-Alkyl: PHE306
2	.alpha.-Phellandrene	−6.0	Pi-Alkyl: PHE273, PHE306
3	Camphene	−5.5	Pi-Sigma: PHE273, PHE306 Pi-Alkyl: PHE306
4	.beta.-Pinene	−5.4	Pi-Sigma: PHE273 Pi-Alkyl: PHE306
5	3-Carene	−6.1	Nil
6	Thujene	−5.5	Alkyl/Pi-Alkyl: LEU70, TYR170
7	.beta.-Myrcene	−4.9	Alkyl: CYS37, LEU70
8	Limonene	−5.8	Alkyl: CYS37, LEU70, ILE200
9	Eucalyptol	−5.4	Pi-Alkyl: PHE273, PHE306
10	p-Cymene	−6.0	Pi-Pi Stacked/Shaped: PHE273, PHE306 Pi-Alkyl: PHE306
11	Methyl-4,6-decadienyl ether	−5.1	H: THR75 C-H: ASP177 Alkyl/Pi-Alkyl: TYR36, CYS37, LEU70
12	Copaene	−7.0	Pi-Alkyl: PHE273, PHE306
13	.alpha.-Bourbonene	−6.5	Pi-Sigma: PHE273, PHE306 Alkyl/Pi-Alkyl: LYS305
14	Acetic acid, octyl ester	−5.0	H: GLN174 Alkyl/Pi-Alkyl: TYR36, CYS37, LEU70
15	Linalool	−5.3	H: THR75, TYR170 Alkyl: CYS37, ILE200
16	Linalool acetate	−5.5	H: ASP40 Alkyl/Pi-Alkyl: TYR36, LEU70
17	Menthyl acetate	−6.2	H: CYS37
18	Caryophyllene	−6.8	Pi-Sigma: PHE273 Pi-Alkyl: PHE306
19	p-Menthan-3-one, cis--	−5.9	Nil
20	Geranyl acetate	−5.8	H: THR75, GLN174 Alkyl/Pi-Alkyl: ALA39, HIS50
21	Bornyl acetate	−5.8	H: ASP40, GLN174 Alkyl: ALA39
22	Isomenthone	−5.4	Alkyl: CYS37, ILE200
23	alpha-terpineol	−6.0	H: GLN190 Alkyl: LEU70
24	gamma.-Cadinene	−7.4	Alkyl/Pi-Alkyl: CYS37, TYR36, ILE200
25	p-menth-1-en-8-ol	−6.5	H: TRY170, ASP40 Alkyl: CYS37
26	p-Cymen-8-ol	−6.4	H: TYR170 Alky/Pi-Alkyl: TYR36, LEU70
27	Benzenemethanol, .alpha., .alpha., 4-trimethyl-	−6.5	H: TYR170 Alkyl: CYS37
28	Verbenone	−5.8	Nil
29	beta.-Elemene	−6.5	Pi-Sigma: PHE306 Alkyl/Pi-Alkyl: PHE273, LYS305
30	Caryophyllene oxide	−6.8	Nil
31	.tau.-Cadinol	−6.9	Unfavorable Donor-Donor: GLY193 Alkyl: CYS37
32	1,3 hexadiene, 3-ethyl, 2,5-dimethyl-	−5.1	Alkyl: CYS37
33	Verticiol	−6.8	H: GLU302
34	Eugenol	−6.4	H: ASP177 Alkyl/Pi-Alkyl: CYS37, LEU70, ILE200
35	2-Propen-1-ol, 3-phenyl-, acetate, (E)-	−3.2	H: THR75 Alkyl/Pi-Alkyl: LEU70, TYR36
36	Cinnamaldehyde, (E)-	−5.8	H: ARG58 Pi-Pi Stacked/Shaped: PHE273, PHE306

**Table 11:** Showing the binding energies and bonding interaction between the chemical compounds of *Boswellia* oil and DNA gyrase protein (PDB:1AB4).

S/No	Chemical Compound of the Frankincense Oil	Binding Energies (kcal/mol)	Bonding Interaction (1AB4 + Compound)
1	.alpha.-Pinene	−5.1	Alkyl/Pi-Alkyl: TYR100, ILE130
2	.alpha.-Phellandrene	−5.2	Alkyl: MET101, ALA128, ILE130
3	Camphene	−5.0	Nil
4	.beta.-Pinene	−5.2	Nil
5	3-Carene	−5.3	Alkyl: ALA128, LYS129, ILE130
6	Thujene	−5.1	Alkyl: MET101, ALA128, ILE130
7	.beta.-Myrcene	−4.8	Alkyl/Pi-Alkyl: TRP59, TYR100, ALA128, LYS129, ILE130, PHE513
8	L-Limonene	−5.1	Alkyl/Pi-Alkyl: TYR100, ALA128, ILE130, PHE513
9	D-Limonene	−5.2	Alkyl/Pi-Alkyl: TYR100, ALA128, ILE130, PHE513
9	Eucalyptol	−5.1	Nil
10	p-Cymene	−5.2	Pi-Sulfur: MET101 Alkyl/Pi-Alkyl: TYR100, ALA128, ILE130, PHE513
11	Methyl-4,6-decadienyl ether	−4.4	Alkyl/Pi-Alkyl: TYR100, ALA128, LYS129, ILE130, PHE513
12	Copaene	−6.2	Alkyl: MET101, ALA128
13	.alpha.-Bourbonene	−6.4	Alkyl/Pi-Alkyl: ALA128, LYS129, ILE130, PHE513
14	Acetic acid, octyl ester	−4.4	Alkyl/Pi-Alkyl: TYR100, ALA128, ILE130, PHE513
15	Linalool	−4.6	H: ILE130 Alkyl/Pi-Alkyl: TYR100, PHE513
16	Linalool acetate	−4.9	H: LYS129 Alkyl/Pi-Alkyl: TYR100, ILE130, PHE513
17	Menthyl acetate	−5.2	Alkyl: MET101, ALA128
18	(*E*)-β-Caryophyllene	−6.1	Nil
19	p-Menthan-3-one, cis-	−5.0	Nil
20	Geranyl acetate	−5.3	Alkyl/Pi-Alkyl:ILE130, PHE513
21	Bornyl acetate	−5.5	H: LYS129 Alkyl: ILE130
22	Isomenthone	−5.3	H: LYS129, ILE130 Alkyl/Pi-Alkyl: TYR100, PHE513
23	alpha-terpinenol	−5.2	H: GLN267 Pi-Alkyl: PHE96
24	gamma.-Cadinene	−6.2	Alkyl/Pi-Alkyl: TYR100, ALA128, LYS129, ILE130, PHE513
25	p-menth-1-en-8-ol	−5.3	H: GLY114 Pi-Alkyl: TYR266, PHE96
26	Carvone	−5.5	H: MET101 Alkyl: ALA128
27	p-Cymen-8-ol	−5.4	Pi-Sulfur: MET101 Alkyl/Pi-Alkyl: TYR100, ALA128, ILE130, PHE513
28	Verbenone	−5.5	Van der Waals: MET101, PHE513 C-H: TYR100 Alkyl: ILE130
29	beta.-Elemene	−5.9	Alkyl: ALA128, LYS129
30	Caryophyllene oxide	−6.3	Nil
31	.tau.-Cadinol	−6.1	Alkyl/Pi-Alkyl: TYR100, ALA128, LYS129, ILE130, PHE513
32	Verticiol	−7.1	H: MET101
33	Eugenol	−5.2	H: LYS129 Pi-Sulfur: MET101 Alkyl/Pi-Alkyl: ALA128, ILE130, PHE513
34	Cinnamyl acetate	−5.6	H: LYS129 C-H: ASP104 Pi-Sigma: ILE130 Pi-Alkyl: ALA128
35	Cinnamaldehyde, (E)-	−5.2	H: THR219, GLN267 C-H: VAL268 Pi-Pi T-Shaped: PHE96

**Table 12:** Showing the binding energies and bonding interaction between the chemical compounds *Boswellia* oil and peptide deformylase protein (PDB: 1IX1).

S/No	Chemical Compound of the Frankincense Oil	Binding Energies (kcal/mol)	Bonding Interaction (1IX1 + Compound)
1	.alpha.-Pinene	−5.1	Nil
2	.alpha.-Phellandrene	−5.4	Alkyl/Pi-Alkyl: ILE45, CYS131, HIS134
3	Camphene	−5.1	Nil
4	.beta.-Pinene	−5.2	Nil
5	3-Carene	−5.0	Pi-Alkyl: PHE120
6	Thujene	−5.1	Alkyl/Pi-Alkyl: LEU127, VAL130, CYS131, HIS134
7	.beta.-Myrcene	−4.9	Alkyl/Pi-Alkyl: ILE45, TYR88, LEU93, VAL130, HIS134
8	L-Limonene	−5.4	Alkyl/Pi-Alkyl: ILE45, LEU93, CYS131, HIS134
9	Eucalyptol	−5.1	Pi-Sigma: PHE120 Alkyl/Pi-Alkyl: ARG71
10	p-Cymene	−5.5	Pi-Pi Stacked: PHE120 Alkyl/Pi-Alkyl: ARG71, PHE73
11	Methyl-4,6-decadienyl ether	−4.7	Alkyl/Pi-Alkyl: ILE45, LEU93, VAL130, CYS131, HIS134
12	Copaene	−6.2	Alkyl: ILE45, LEU93
13	.alpha.-Bourbonene	−6.9	Alkyl: ILE45, CYS131
14	Acetic acid, octyl ester	−4.8	C-H: GLU135 Alkyl/Pi-Alkyl: TYR88, CYS131, HIS134
15	Linalool	−5.6	H: GLY46 Alkyl/Pi-Alkyl: ILE45, LEU127, VAL130, CYS131, HIS134
16	Linalool acetate	−5.1	H: VAL72, ARG115 Pi-Sigma: PHE120 Alkyl/Pi-Alkyl: ARG71, PHE120
17	Menthyl acetate	−5.6	H: GLY91 Alkyl/Pi-Alkyl: ILE45, LEU93, HIS134
18	(*E*)-β-Caryophyllene	−6.7	Alkyl/Pi-Alkyl: ILE45, HIS134
19	p-Menthan-3-one, cis-	−5.5	Nil
20	Geranyl acetate	−5.5	H: ILE45, GLY46 Alkyl/Pi-Alkyl: LEU127, VAL130, CYS131, HIS134
21	Bornyl acetate	−5.3	H: GLY91 C-H: GLY91 Pi-Sigma: HIS134 Alkyl: ILE45
22	Isomenthone	−5.4	H: GLY91 Alkyl/Pi-Alkyl: ILE45, HIS134
23	alpha-terpineol	−5.9	H: GLY91 Pi-Sigma: HIS134 Alkyl: ILE45, LEU93, CYS131
24	gamma.-Cadinene	−7.0	Alkyl/Pi-Alkyl: ILE45, LEU93, TYR99, CYS131, HIS134
25	p-menth-1-en-8-ol	−6.1	H: GLY46 Alkyl/Pi-Alkyl: ILE45, LEU127, VAL130, CYS131, HIS134
26	Carvone	−5.6	Alkyl/Pi-Alkyl: VAL130, CYS131, HIS134
27	p-Cymen-8-ol	−5.7	Pi-Sigma: ILE45 Pi-Pi Stacked: HIS134 Alkyl/Pi-Alkyl: TYR88, LEU127, VAL130, CYS131
28	Verbenone	−5.7	Nil
29	beta.-Elemene	−6.2	Alkyl/Pi-Alkyl: ILE45, HIS134
30	Caryophyllene oxide	−6.6	Nil
31	.tau.-Cadinol	−7.6	Unfavorable Donor-Donor: ILE45 Alkyl/Pi-Alkyl: TYR88, VAL130, CYS131, HIS134
32	Verticiol	−6.4	Unfavorable Donor-Donor/Unfavorable Acceptor-Acceptor: ARG71, GLU122
33	Eugenol	−5.8	H: ILE45, GLY46 Pi-Pi Stacked: HIS134 Alkyl/Pi-Alkyl: LEU93, LEU127, VAL130, CYS131
34	Cinnamyl acetate	−5.8	H: CYS92, LEU93 Pi-Sigma: ILE45 Alkyl: CYS131
35	Cinnamaldehyde, (E)-	−5.6	H: GLN51, CYS92, LEU93 C-H: GLY46 Pi-Sigma: ILE45 Pi-Alkyl: CYS131

**Table 13:** Showing the binding energies and bonding interaction between the chemical compounds of *Boswellia* oil and 1,3-β-glucan synthase protein (PDB: 7XE4).

S/No	Chemical Compound of the Frankincense Oil	Binding Energies (Kcal/mol)	Bonding Type (7XE4 + Compound)
1	.alpha.-Pinene	−6.3	Pi-Sigma: PHE1366 Alkyl/Pi-Alkyl: ILE1304, LEU1308, ILE1362, PHE1363
2	.alpha.-Phellandrene	−5.8	Alkyl/Pi-Alkyl: TRP515, LEU528, PHE532
3	Camphene	−6.1	Van der Waals: PHE1363 Pi-Sigma: PHE1366 Alkyl/Pi-Alkyl: LEU1308, ILE1362
4	.beta.-Pinene	−6.4	Alkyl/Pi-Alkyl: ILE1304, LEU1308, ILE1362, PHE1363, PHE1366
5	3-Carene	−6.2	Alkyl/Pi-Alkyl: LEU1308, PHE1363, PHE1366
6	Thujene	−6.0	Alkyl/Pi-Alkyl: LEU1308, PHE1363, PHE1366
7	.beta.-Myrcene	−4.8	Alkyl/Pi-Alkyl: ILE1340, LEU1308, ILE1362, PHE1366, PHE1370
8	L-Limonene	−6.0	Alkyl/Pi-Alkyl: ILE1304, LEU1308, ILE1362, PHE1363, PHE1366
9	Eucalyptol	−5.8	Van der Waals: ILE1304, ILE1362, PHE1363, PHE1366, PHE1475
10	p-Cymene	−6.1	Pi-Cation: ARG1273 Pi-Sigma: HIS1218 Amide-Pi Stacked: GLY1216 Pi-Alkyl: LEU1217
11	Methyl-4,6-decadienyl ether	−4.5	H: SER1478 Alkyl/Pi-Alkyl: ILE1304, LEU1308, ILE1362, PHE1370
12	Copaene	−7.3	Pi-Sigma: PHE1366 Alkyl/Pi-Alkyl: ILE1304, PHE1370
13	.alpha.-Bourbonene	−7.6	Pi-Sigma: PHE1366 Alkyl/Pi-Alkyl: LEU1308
14	Acetic acid, octyl ester	−4.3	Alkyl/Pi-Alkyl: LEU1308, ILE1304, PHE1366, PHE1363, ILE1362
15	Linalool	−4.9	Alkyl/Pi-Alkyl: ILE1304, PHE1366, PHE1370
16	Linalool acetate	−5.4	H: SER1478 Alkyl/Pi-Alkyl: LEU1308, ILE1362, PHE1363, PHE1366
17	Menthyl acetate	−6.3	H: SER1478 Alkyl/Pi-Alkyl: ILE1304, LEU1308, ILE1362, PHE1363, PHE1475
18	(*E*)-β-Caryophyllene	−7.4	Nil
19	p-Menthan-3-one, cis-	−5.7	H: SER1478 Pi-Sigma: PHE1366
20	Geranyl acetate	−5.8	H: LYS1212, TYR1224, ASN1228 C-H: GLY1208 Alkyl/Pi-Alkyl: TRP398, LEU1231
21	Bornyl acetate	−6.4	Pi-Sigma: PHE1366 Alkyl/Pi-Alkyl: ILE1304, LEU1308
22	Isomenthone	−5.7	H: SER1478 Pi-Sigma: PHE1366
23	alpha-terpinenol	−5.7	Alkyl: ILE1304, LEU1308
24	gamma.-Cadinene	−7.4	Alkyl/Pi-Alkyl: ILE1304, LEU1308, ILE1362, PHE1366
25	p-menth-1-en-8-ol	−6.1	H: ARG1273 Unfavorable Donor-Donor: ASP392 Pi-Alkyl: TYR439
26	Carvone	−6.2	Pi-Alkyl: PHE532
27	p-Cymen-8-ol	−5.7	Pi-Pi Stacked: PHE1176 Pi-Alkyl: PHE1176
28	Verbenone	−6.2	Nil
29	beta.-Elemene	−7.1	Alkyl/Pi-Alkyl: ILE1304, LEU1308, ILE1362, PHE1363, PHE1366, PHE1475
30	Caryophyllene oxide	−7.2	Nil
31	tau.-Cadinol	−7.2	Pi-Sigma: PHE1366 Alkyl/Pi-Alkyl: LEU1308, PHE1363, LEU1479, LEU1482
32	Verticiol	−7.3	Pi-Sigma: PHE629 Alkyl: MET458
33	Eugenol	−6.5	H: ILE387, GLY389, LYS437 C-H: HIS384, GLU441 Pi-Cation/Pi-Anion: ASP392, ARG1273 Amide-Pi Stacked: GLY1216 Alkyl/Pi-Alkyl: LEU1217
34	Cinnamyl acetate	−5.6	H: GLN604 C-H: GLN604 Pi-Sigma/Pi-Pi T-shaped: PHE610 Alkyl/Pi-Alkyl: ALA608, PRO1283
35	Cinnamaldehyde, (E)-	−5.5	Pi-Sigma: PHE532 Pi-Pi Stacked: TRP515 Pi-Alkyl: LEU528

## Data Availability

The report of the analysis performed for the samples in the paper can be found at the Interdisci-plinary Research Platform (PCI) belonging to the University of Life Sciences “King Michael I of Romania” from Timisoara.

## Supplementary Materials

The following supporting information can be downloaded at: https://www.mdpi.com/article/10.3390/antiox12101807/s1, Supplementary file S1: Chemical composition of the tested frankincense essential oil detected by GC-MS; Supplementary file S2 Figure S1: 3D and 2D display of some of the interaction between the enantiomers of the chemical compounds from Boswellia oil and bacterial TyrRS; Supplementary file S2 Figure S2: 3D and 2D display of some of the interaction between the enantiomers of the chemical compounds from Boswellia oil and fungal 1,3-β-glucan synthase; Supplementary file S2 Figure S3: 3D and 2D display of some of the interaction between the enantiomers of the chemical compounds from Boswellia oil and bacterial DNA gyrase; Supplementary file S2 Figure S4: 3D and 2D display of some of the interaction between the enantiomers of the chemical compounds from Boswellia oil and bacterial peptide deformylase; Supplementary file S2 Table S1: Interaction of the enantiomers of some chemical compounds from Boswellia oil with the bacterial TyrRS; Supplementary file S2 Table S2: Interaction of the enantiomers of some chemical compounds from Boswellia oil with the fungal 1,3-β-glucan synthase; Supplementary file S2 Table S3: Interaction of the enantiomers of some chemical compounds from Boswellia oil with the bacterial DNA gyrase; Supplementary file S2 Table S4: Interaction of the enantiomers of some chemical compounds from Boswellia oil with the bacterial peptide deformylase.
