# Supplementary material for: Boswellia Essential Oil: Natural Antioxidant as an Effective Antimicrobial and Anti-Inflammatory Agent
Source: Antioxidants (Basel). 2023 Sep 27;12(10):1807. doi: 10.3390/antiox12101807 (PMC10603989; doi:10.3390/antiox12101807)
Supplement: Supplementary file 1 [file antioxidants-12-01807-s001.zip › Supplementary file S1.pdf]

Chemical composition of the tested frankincense essential oil detected by GC-

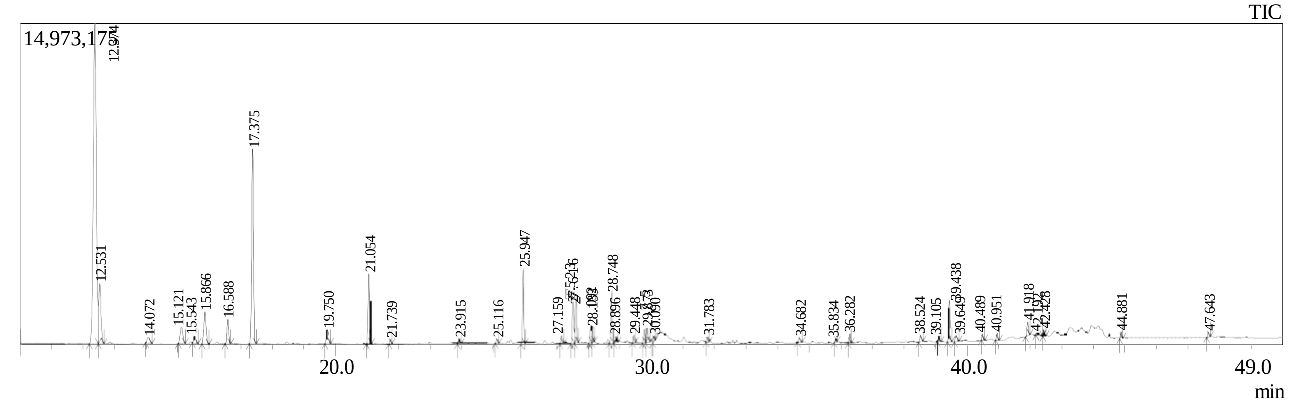

Quantitative Result Table

ID#	Name	R.Time	m/z	Area	Height	Conc.
1	.alpha.-Pinene	12.374	TIC	100975318	14911684	39.34
2	.alpha.-Phellandrene	12.531	TIC	14035230	2821573	5.48
3	Camphene	14.072	TIC	2214014	323220	0.77
4	.beta.-Pinene	15.121	TIC	4838912	788419	1.89
5	3-Carene	15.543	TIC	1713604	358685	0.67
6	Thujene	15.866	TIC	7676176	1503896	3.00
7	.beta.-Myrcene	16.588	TIC	4448378	1159333	1.74
8	Limonene	17.375	TIC	35391255	9071728	13.79
9	Eucalyptol	19.750	TIC	3227237	678612	1.21
10	p-Cimene	21.054	TIC	10768301	3268943	4.21
11	Methyl 4,6-decadienyl ether	21.739	TIC	615303	218897	0.24
12	Copaene	23.915	TIC	1004088	247989	0.39
13	.alpha.-Bourbonene	25.116	TIC	790325	224944	0.33
14	Acetic acid, octyl ester	25.947	TIC	9494968	3522649	3.71
15	tau-Elemene	27.159	TIC	1290198	380552	0.51
16	Linalool	27.523	TIC	6322287	1961389	2.47
17	Linalool acetate	27.616	TIC	6571945	2105615	2.58
18	Menthyl acetate	28.092	TIC	5186571	858045	2.03
19	Caryophyllene	28.092	TIC	5186571	858045	2.03
20	Isomenthone	28.748	TIC	7302245	2452804	2.86
21	Geranyl acetate	28.896	TIC	929487	319979	0.40
22	Bornyl acetate	29.448	TIC	1887741	360891	0.75
23	Menthone	29.775	TIC	1859332	637164	0.73
24	4-Terpinenol	29.873	TIC	2465736	689143	0.97
25	gamma.-Cadinene	30.090	TIC	564358	230047	0.24
26	p-menth-1-en-8-ol	31.783	TIC	1250452	300240	0.46
27	Carvona	34.682	TIC	1223310	242922	0.47
28	p-Cymen-8-ol	35.834	TIC	770022	215318	0.31
29	Verbenone	36.282	TIC	1294327	454547	0.51
30	beta.-Elemene	38.524	TIC	1288665	343251	0.50
31	Caryophyllene oxide	39.438	TIC	5785928	1949184	2.26
32	.tau.-Cadinol	39.649	TIC	1251460	291428	0.47
33	Verticiol	40.951	TIC	1165577	351850	0.46
34	Eugenol	41.918	TIC	2509467	706809	0.98
35	Cinnamyl acetate	42.201	TIC	1204282	179488	0.48
36	Cinnamaldehyde, (E)-	42.428	TIC	854994	310503	0.38

*Residual non-natural compounds represent the difference of 0.41%.
