# Supplementary material for: Boswellia Essential Oil: Natural Antioxidant as an Effective Antimicrobial and Anti-Inflammatory Agent
Source: Antioxidants (Basel). 2023 Sep 27;12(10):1807. doi: 10.3390/antiox12101807 (PMC10603989; doi:10.3390/antiox12101807)
Supplement: Supplementary file 1 [file antioxidants-12-01807-s001.zip › Supplementary file S2.pdf]

Table S1. Interaction of the enantiomers of some chemical compounds from *Boswellia* oil with the bacterial TyrRS

S/No	Enantiomers of chemicals compounds from frankincense oil	Binding energies (kcal/mol)	Binding interaction (1JIJ + compound)
1	(-)-Camphene	-5.5	**Pi-Alkyl:** PHE273, PHE306
2	(+)-Camphene	-5.6	Nil
3	(+)-Linalyl acetate	**-5.8**	**H:** ASN124
4	Linalyl acetate, (-)-	**-5.2**	**H:** PHE273<br>**Pi-Sigma:** PHE273<br>**Pi-Alkyl:** PHE306
5	alpha-Pinene, (-)-	-8.2	**Pi-Alkyl:** PHE273, PHE306
6	alpha-Pinene, (+)-	-8.2	Nil
7	beta-Pinene, (-)-	-8.2	Nil
8	beta-Pinene, (+)-	-8.2	Nil
9	Linalool, (-)-	**-5.1**	**H:** TYR170, GLN174<br>**Alkyl/Pi-Alkyl:** TYR36, LEU70
10	Linalool, (+)-	**-5.4**	**H:** THR75<br>**Alkyl:** CYS37
11	D-Limonene	-5.8	**Alkyl:** CYS37, LEU70, ILE200

Figure S1. 3D and 2D display of some of the interaction between the enantiomers of the chemical compounds from *Boswellia* oil and bacterial TyrRS

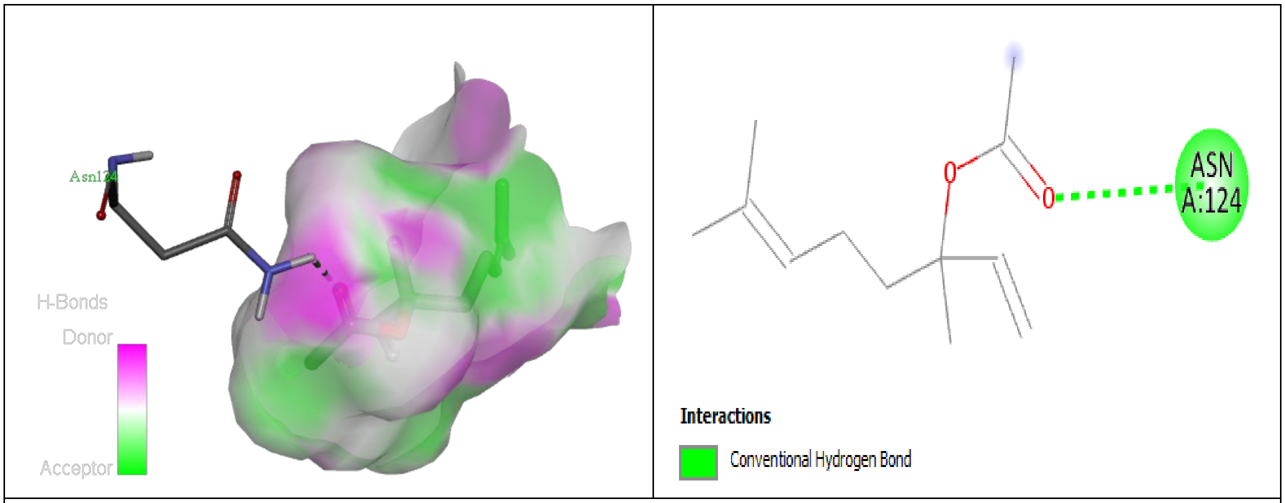

(+)-Linalyl acetate – 1JIJ

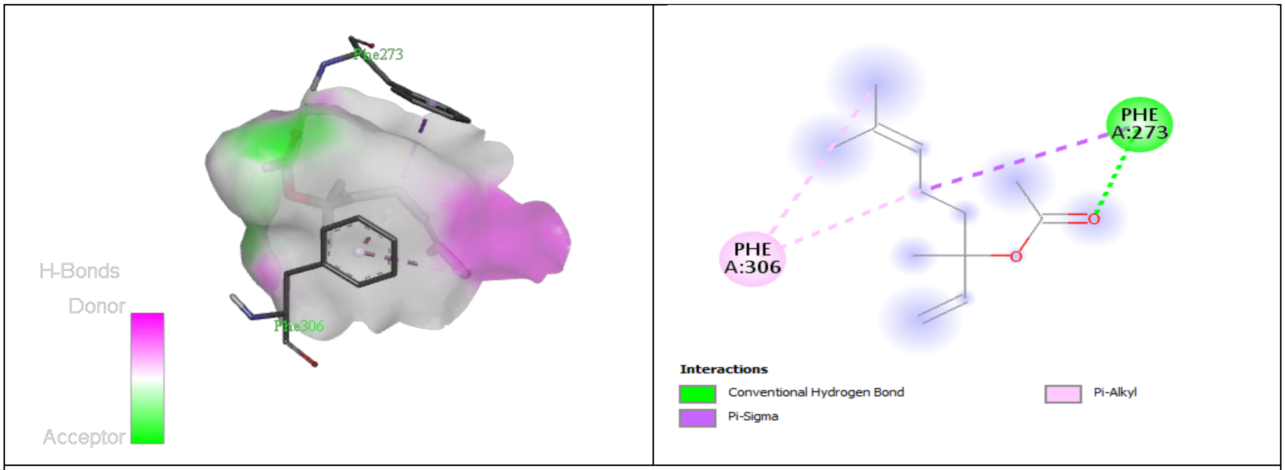

Linalyl acetate, (-)- - 1JIJ

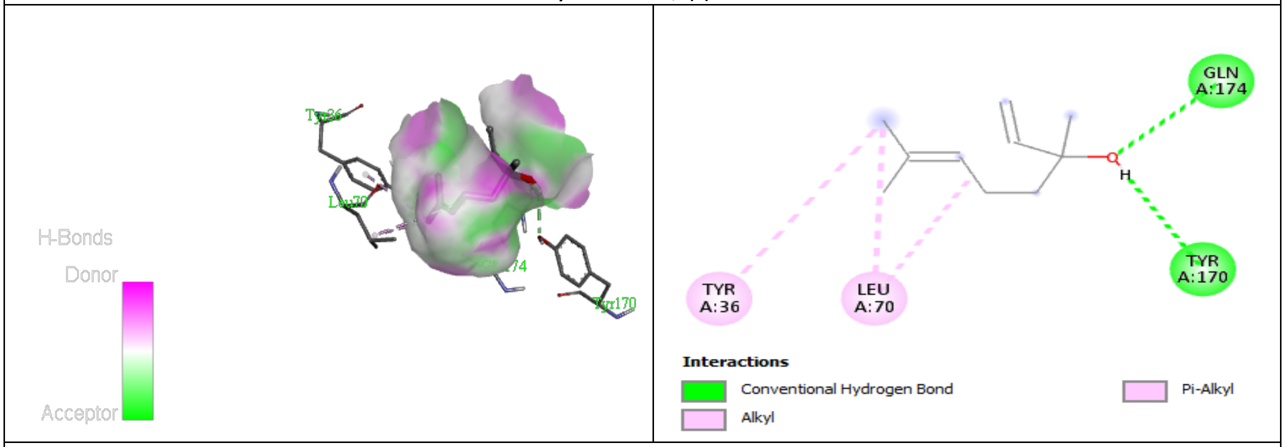

Linalool, (-)- - 1JIJ

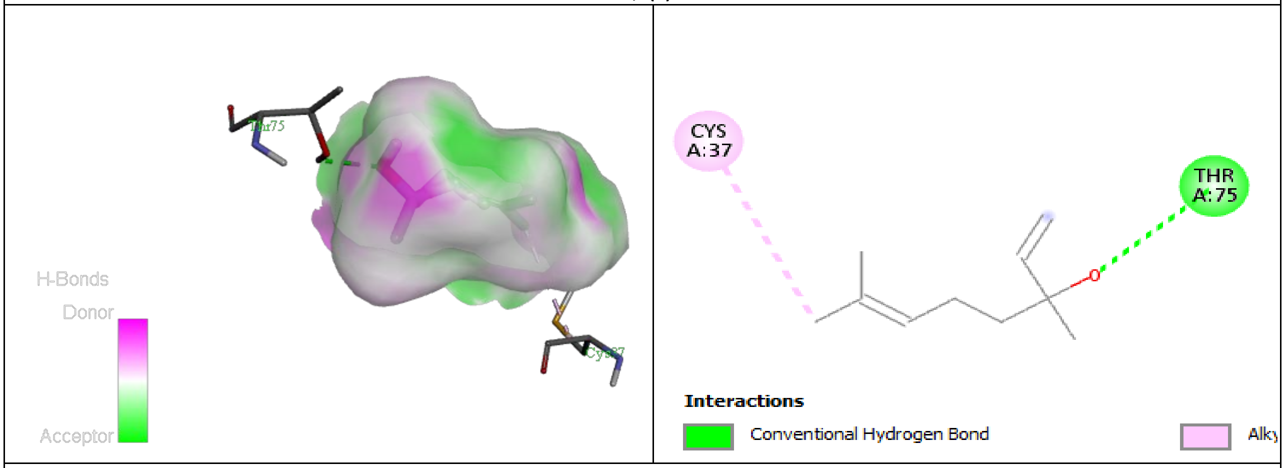

Linalool, (+)- - 1JIJ

Table S2. Interaction of the enantiomers of some chemical compounds from *Boswellia* oil with the fungal 1,3-β-glucan synthase

S/No	Enantiomers of chemicals compounds from frankincense oil	Binding energies (kcal/mol)	Binding interaction (7XE4 + compound)
1	(-)-Camphene	**-6.1**	Van der Waals: ILE1362, ILE1363<br>Alkyl/Pi-Alkyl: LEU1308, PHE1366
2	(+)-Camphene	**-6.3**	Van der Waals: PHE1363<br>Alkyl/Pi-Alkyl: ILE1304, LEU1308, ILE1362, PHE1366
3	(+)-Linalyl acetate	-5.7	Pi-Sigma: PHE1366<br>Alkyl: ILE1304, LEU1308
4	Linalyl acetate, (-)-	**-5.3**	H: SER1478<br>Alkyl/Pi-Alkyl: ILE1304, LEU1308, ILE1362, PHE1366, PHE1475
5	alpha-Pinene, (-)-	-8.3	Nil
6	alpha-Pinene, (+)-	-8.4	Nil
7	beta-Pinene, (-)-	-8.4	Nil
8	beta-Pinene, (+)-	-8.4	Nil
9	Linalool, (-)-	-4.7	Alkyl/Pi-Alkyl: ILE1304, PHE1366, PHE1370
10	Linalool, (+)-	-4.9	Alkyl/Pi-Alkyl: ILE1304, LEU1308, PHE1363, PHE1366, PHE1370, PHE1475, LEU1479
11	D-Limonene	-5.8	Pi-Sigma: PHE1366<br>Alkyl/Pi-Alkyl: ILE1304, LEU1308, ILE1362, PHE1363

Figure S2. 3D and 2D display of some of the interaction between the enantiomers of the chemical compounds from *Boswellia* oil and fungal 1,3-β-glucan synthase

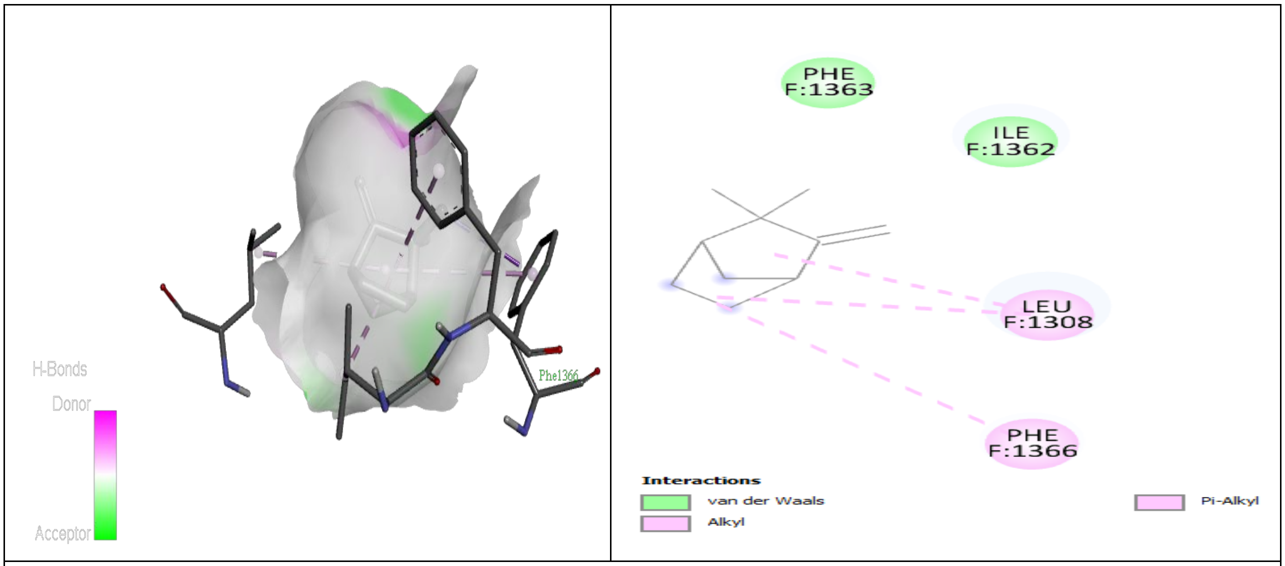

(-)-Camphene – 7XE4

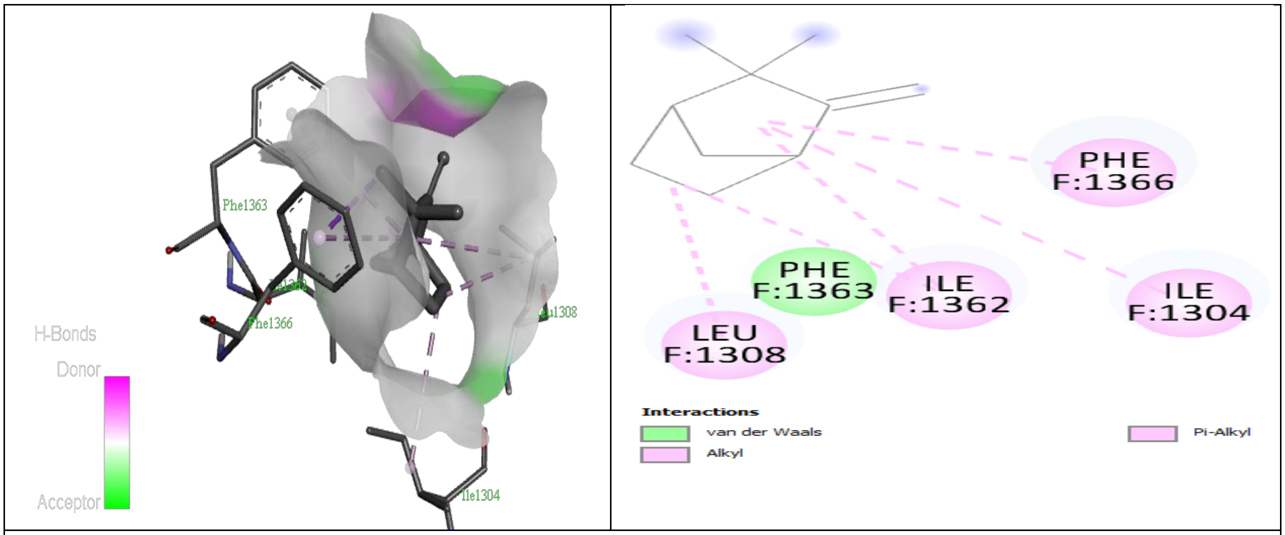

(+)-Camphene – 7XE4

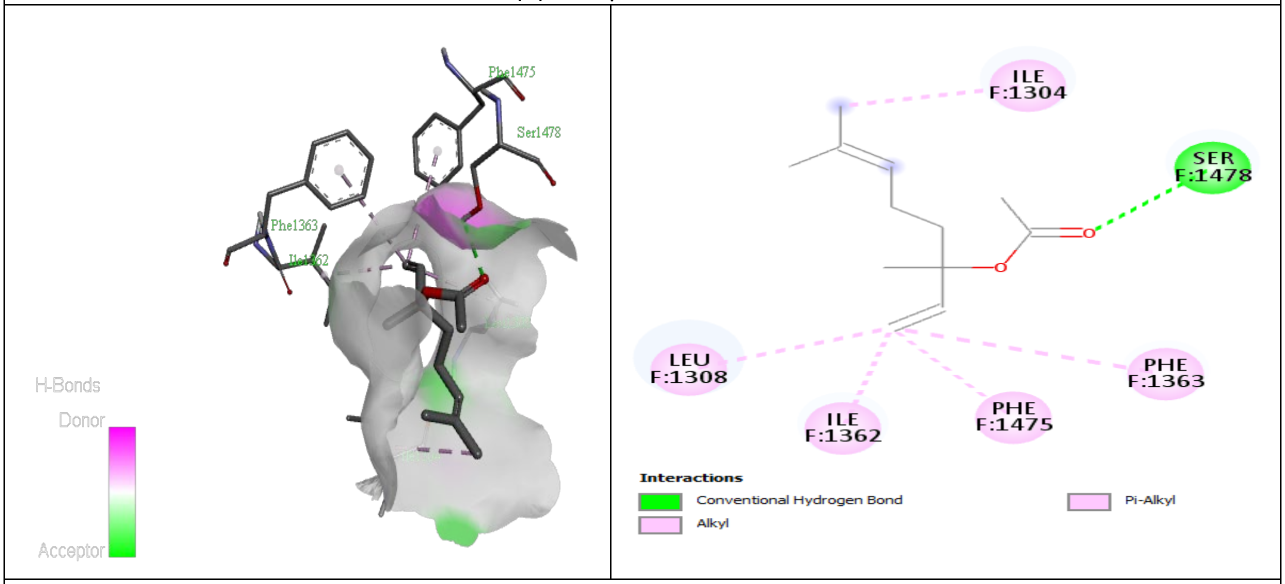

Linalyl acetate, (-)- - 7XE4

Table S3. Interaction of the enantiomers of some chemical compounds from *Boswellia* oil with the bacterial DNA gyrase

S/No	Enantiomers of chemicals compounds from frankincense oil	Binding energies (kcal/mol)	Binding interaction (1AB4 + compound)
1	(-)-Camphene	**-5.0**	Van der Waals: TYR100<br>Alkyl/Pi-Alkyl: ILE130, PHE513
2	(+)-Camphene	-5.6	Nil
3	(+)-Linalyl acetate	**-4.7**	H: LYS129<br>Alkyl/Pi-Alkyl: TYR100, ALA128, ILE130, PHE513

4	Linalyl acetate, (-)-	**-5.0**	C-H: PHE513<br>Alkyl: ALA128, LYS129, ILE130
5	alpha-Pinene, (-)-	-6.7	Nil
6	alpha-Pinene, (+)-	-8.0	Nil
7	beta-Pinene, (-)-	-8.0	Nil
8	beta-Pinene, (+)-	-8.0	Nil
9	Linalool, (-)-	-4.7	Pi-Alkyl:TYR266
10	Linalool, (+)-	**-4.9**	H: GLU369<br>Alkyl: LEU488
11	D-Limonene	-5.2	Alkyl/Pi-Alkyl: TYR100, ALA128, ILE130, PHE513

Figure S3. 3D and 2D display of some of the interaction between the enantiomers of the chemical compounds from *Boswellia* oil and bacterial DNA gyrase

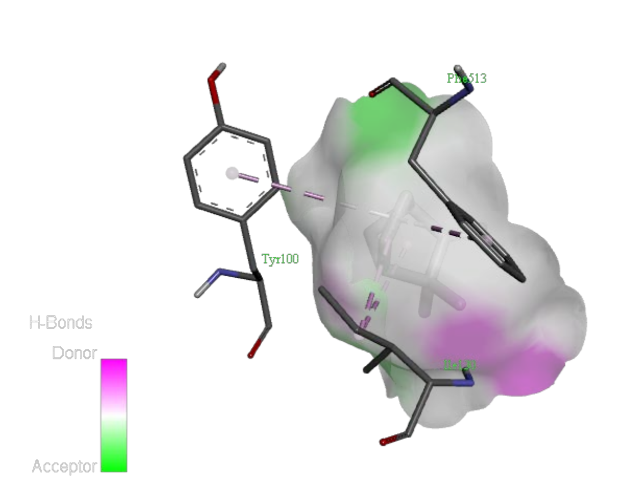

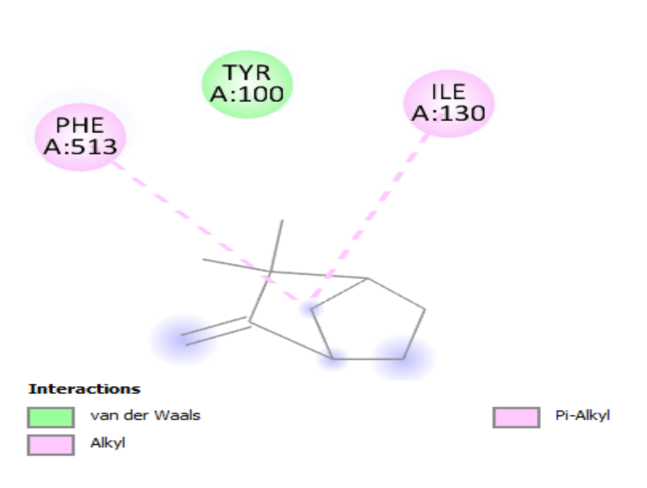

(-)-Camphene – 1AB4

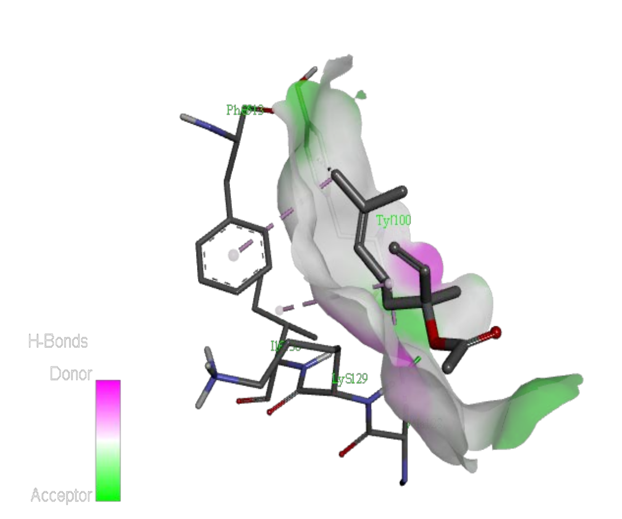

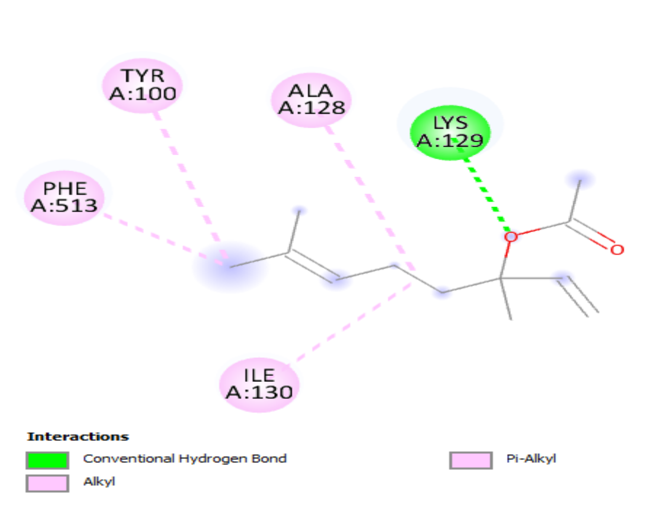

(+)-Linalyl acetate – 1AB4

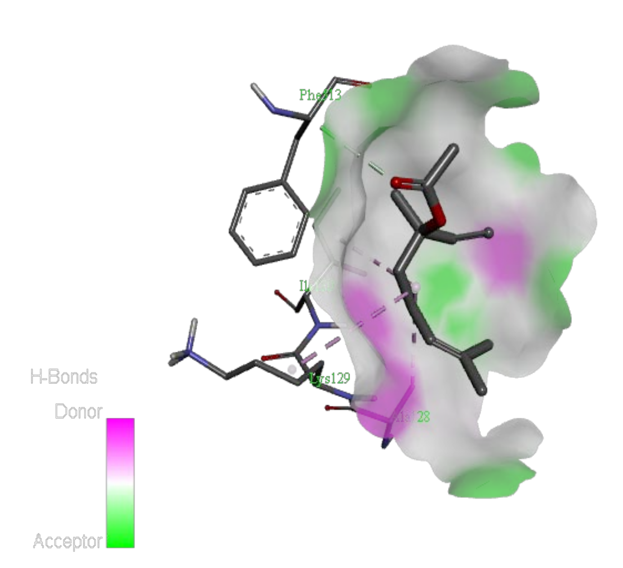

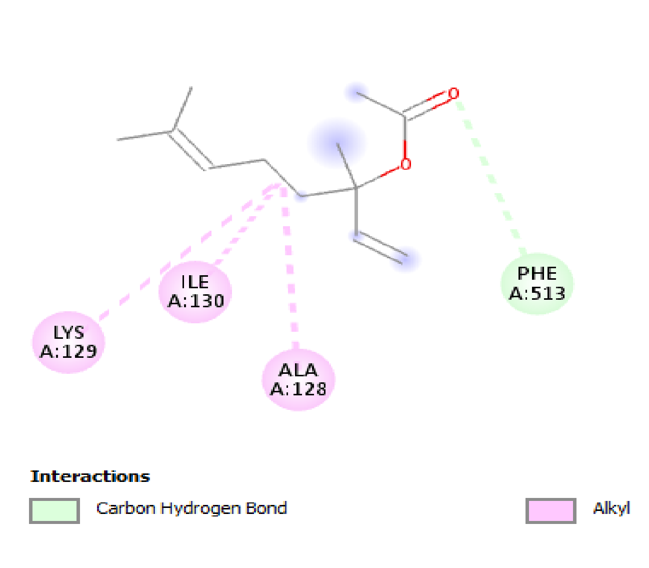

Linalyl acetate, (-)- - 1AB4

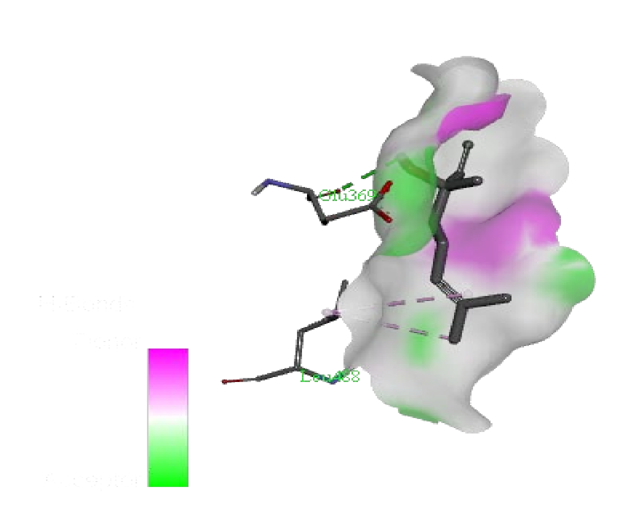

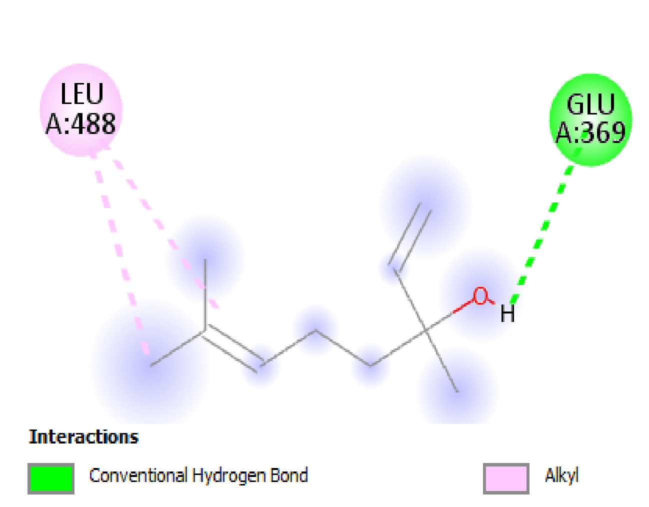

Linalool, (+)- - 1AB4

Table S4. Interaction of the enantiomers of some chemical compounds from *Boswellia* oil with the bacterial peptide deformylase

S/No	Enantiomers of chemicals compounds from frankincense oil	Binding energies (kcal/mol)	Binding interaction (1IX1 + compound)
1	(-)-Camphene	-5.1	Nil
2	(+)-Camphene	-5.3	Nil
3	(+)-Linalyl acetate	**-5.3**	H: ILE45, GLY46<br>C-H: GLY44<br>Alkyl/Pi-Alkyl: TYR88, LEU127, VAL130

4	Linalyl acetate, (-)-	**-5.4**	H: ARG115<br>Alkyl/Pi-Alkyl: ARG71, PHE73,PHE120
5	alpha-Pinene, (-)-	-6.9	Nil
6	alpha-Pinene, (+)-	-6.9	Nil
7	beta-Pinene, (-)-	-6.9	Nil
8	beta-Pinene, (+)-	-6.9	Nil
9	Linalool, (-)-	**-5.7**	H: ILE45, GLY46<br>Alkyl/Pi-Alkyl: CYS92, LEU93, LEU127, VAL130, CYS131, HIS134
10	Linalool, (+)-	-5.6	Unfavorable Donor-Donor: GLY46<br>Alkyl/Pi-Alkyl: ILE45, LEU93, LEU127, VAL130, CYS131, HIS134
11	D-Limonene	-5.4	Alkyl/Pi-Alkyl: ILE45, LEU93, CYS131, HIS134

Figure S4. 3D and 2D display of some of the interaction between the enantiomers of the chemical compounds from *Boswellia* oil and bacterial peptide deformylase

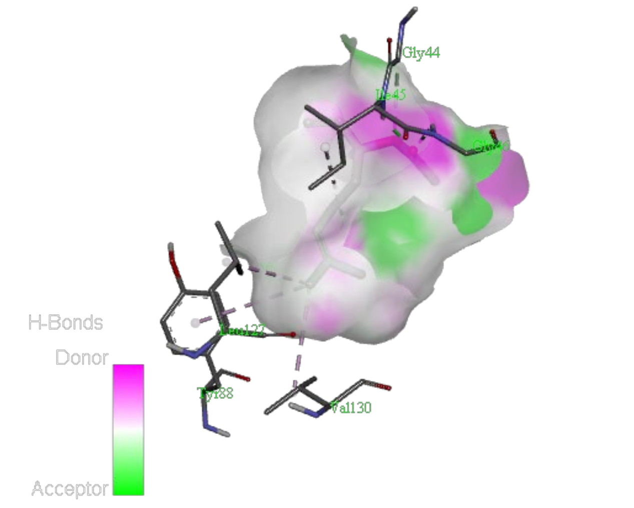

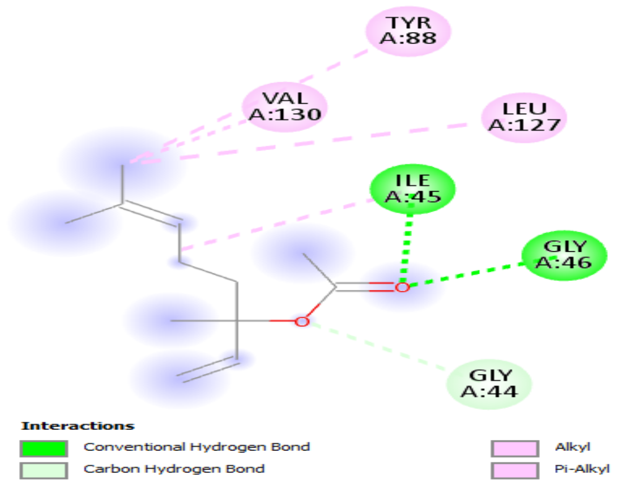

(+)-Linalyl acetate – 1IX1

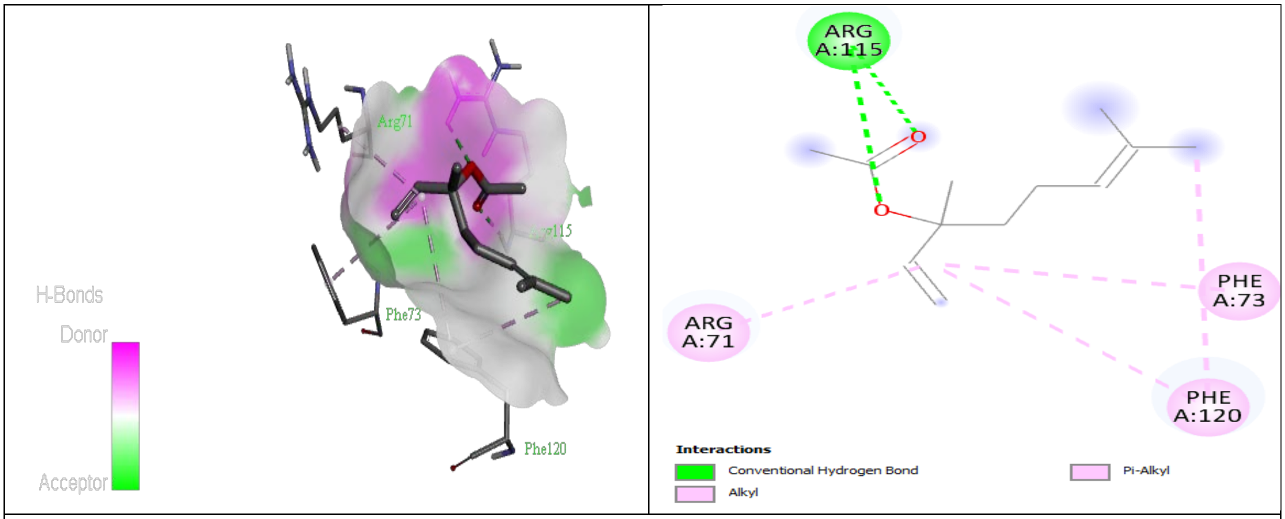

Linalyl acetate, (-)- - 1IX1

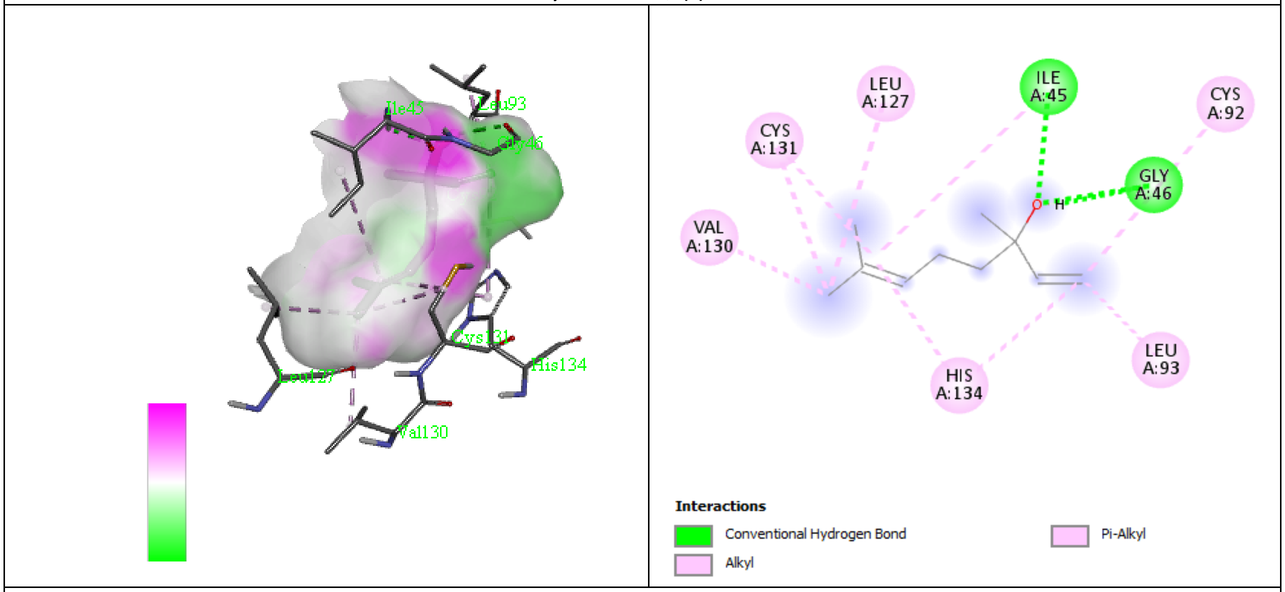

Linalool, (-)- - 1IX1
